# Manipulating vector transmission reveals local processes in *Bartonella* communities of bats

**DOI:** 10.1017/S0031182026101656

**Published:** 2026-03

**Authors:** Clifton D. McKee, Colleen T. Webb, Michael Y. Kosoy, Richard Suu-Ire, Yaa Ntiamoa-Baidu, Andrew A. Cunningham, James L. N. Wood, David T. S. Hayman

**Affiliations:** 1 Department of Epidemiology, Johns Hopkins Bloomberg School of Public Health, Baltimore, MD, USA; 2 Graduate Degree Program in Ecology, Colorado State University, Fort Collins, CO, USA; 3 Department of Biology, Colorado State University, Fort Collins, CO, USA; 4 KB One Health LLC, Fort Collins, CO, USA; 5 School of Veterinary Medicine, University of Ghana, Accra, Ghana; 6 Centre for Biodiversity Conservation Research, University of Ghana, Accra, Ghana; 7 Department of Animal Biology and Conservation Science, University of Ghana, Accra, Ghana; 8 Institute of Zoology, Zoological Society of London, Regent’s Park, London, UK; 9 Disease Dynamics Unit, Department of Veterinary Medicine, University of Cambridge, Cambridge, UK; 10 Molecular Epidemiology and Public Health Laboratory (mEpiLab), Infectious Disease Research Centre, Hopkirk Research Institute, Massey University, Palmerston North, New Zealand

**Keywords:** *Bartonella*, bats, community assembly, ecological dynamics, pathogen diversity, vector-borne bacteria

## Abstract

Infectious diseases result from multiple interactions among microbes and hosts, but community ecology approaches are rarely applied. Manipulation of vector populations provides a unique opportunity to test the importance of vectors in infection cycles while also observing changes in pathogen community diversity and species interactions. Yet for many vector-borne infections in wildlife, a biological vector has not been experimentally verified, and few manipulative studies have been performed. Using a captive colony of fruit bats in Ghana, we conducted the first study to experimentally test the role of bat flies as vectors of *Bartonella* species. We observed changes in the *Bartonella* bacteria community over time following the decline of bat flies and again after their subsequent restocking. Reduced transmission rates led to microbial community changes attributed to ecological drift and potential species sorting through interspecific competition mediated by host immunity. We demonstrate that forces maintaining diversity in communities of free-living macroorganisms act in similar ways in communities of symbiotic microorganisms, both within and among hosts.

## Graphical abstract

**Figure:**
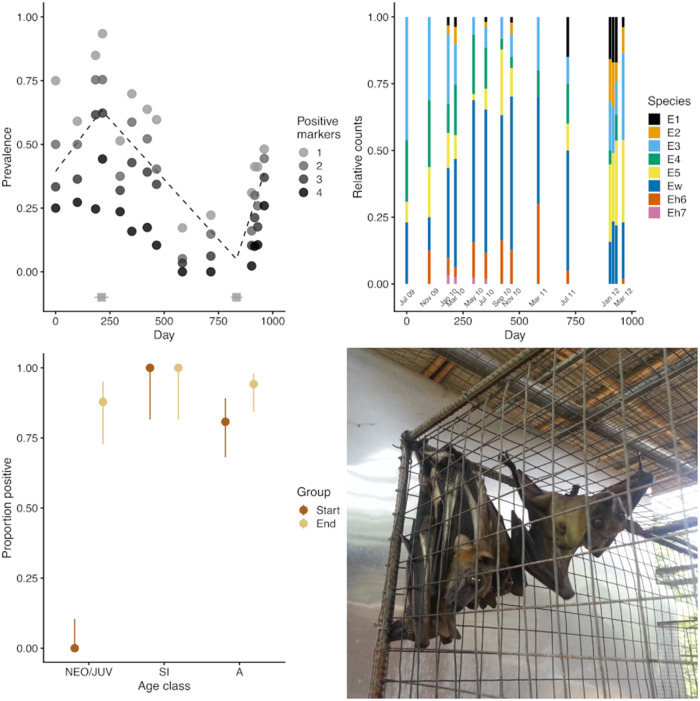

## Introduction

Knowledge of the processes driving parasite diversity is central to understanding infection dynamics in endemic populations and pathogen emergence in new hosts. In contrast to a historical focus on simple one-host, one-parasite systems, there is now greater appreciation that parasites exist within communities of other parasites, harboured by hosts that may vary in their responses to parasitism (Johnson et al., 2015). Yet it is not clear how well ecological theory developed for free-living, sexually reproducing organisms applies to communities of microorganisms (Sutherland et al., 2013). This is especially true for parasites and symbionts due to the environmental feedbacks that exist from their dependence on hosts for survival and reproduction (Costello et al., 2012; Fierer et al., 2012; Miller et al., 2018; Brown et al., 2020; Skičková et al., 2025; Speer et al., 2025). Additionally, parasite community dynamics within hosts may occur at differing timescales compared to transmission among hosts. Given these differences, experimental manipulations of natural parasite communities are needed to explore the generality of community theory across organisms.

**Figure 1.**
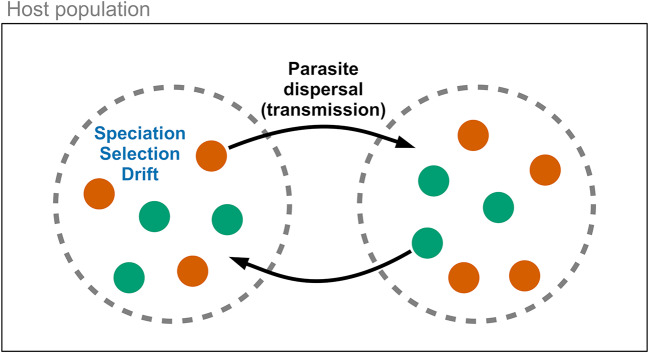
Conceptual diagram for parasite metacommunity dynamics. Parasites species (coloured dots) exist within host populations and disperse among hosts (dashed circles) via transmission. Ecological forces, including speciation, species selection (sorting), and drift, act on parasite communities within host individuals. These processes can be generalized to other ecological scales, such as between hosts and ectoparasitic vectors. Figure 1 long description.

Metacommunities are a valuable framework for analysing parasite community dynamics within hosts (Leibold et al., 2004; Mihaljevic, 2012). In this model, hosts act as discrete patches containing potentially interacting parasite species (Figure 1). Four forces may influence parasite community diversity: speciation, dispersal, ecological drift, and ecological selection (Vellend, 2010). The significance of these forces can vary at different scales (Seabloom et al., 2015), i.e., within versus among hosts. Speciation generates parasite diversity but is generally slow and relies on dispersal for new diversity to spread across scales. Dispersal involves the movement of parasites within a host, among hosts, or among host populations. Within metacommunities, parasite species with equal competitive ability may vary in their production of new individuals, leading to changes in community composition (e.g., loss of rare species or increased beta diversity), similar to neutral theory predictions (Hubbell, 2001). Drift occurs more rapidly in small communities with fewer individuals and limited dispersal. Ecological selection (or species sorting) operates within and among hosts due to variance in replication success influenced by host susceptibility. Parasite species may compete within a host via resource sharing or immune system interactions (Pedersen and Fenton, 2007; Telfer et al., 2010). Species with better success can dominate, but this may be mitigated if fitness is influenced more by dispersal ability than competition, or through frequency-dependent selection by the host immune system. These four forces can independently affect parasite community diversity over time and may vary in their relative importance. However, since dispersal is the force that interacts with other processes across within-host and among-host scales (Vellend, 2010), it is an appealing target for experimental manipulation.

Vector-borne infections are ideal systems for experimental study because the reduction of vector density limits dispersal of parasites between hosts, allowing for the analysis of other forces affecting the relative abundance of parasite species. Our focus in this study is on *Bartonella* bacterial communities in bats and their ectoparasitic flies. *Bartonella* spp. (Alphaproteobacteria: Hyphomicrobiales) are diverse intracellular bacteria that infect mammals and are transmitted by blood-feeding arthropods (Harms and Dehio, 2012). Numerous *Bartonella* species have been recognized as zoonotic pathogens in humans and can cause disease non-human animals (Breitschwerdt, 2014). Host species, including bats, frequently carry multiple distinct *Bartonella* genotypes or species that can vary in their relative abundance over time (Kosoy et al., 2004a; Telfer et al., 2007; Becker et al., 2018; Goodrich et al., 2020; Fagre et al., 2023). Previous studies have proposed that bat flies are vectors of *Bartonella* spp. in bats (Morse et al., 2012; Brook et al., 2015; Moskaluk et al., 2018), but no experimental studies have been performed to demonstrate their competence. Bat flies (Diptera: Streblidae, Nycteribiidae, Mystacinobiidae) are obligate ectoparasites of bat hosts that leave their hosts only briefly for mating and to deposit prepupae in the roost environment (Marshall, 1970; Dick and Patterson, 2006; Dick and Dittmar, 2014). Following these movements, bat flies may return to a new host individual, providing an opportunity for horizontal transmission of *Bartonella* between bats. In this study, we attempt to provide experimental evidence that bat flies are vectors of *Bartonella* by modifying bat fly population density and examining the changes in *Bartonella* prevalence and diversity in bat hosts over time.

**Figure 2.**
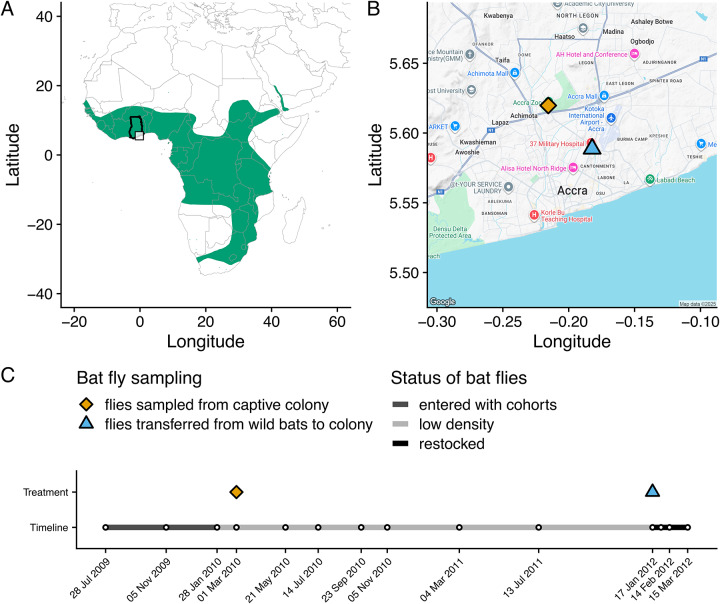
Background information on the study system and experimental design. (A) Map of the geographic range of straw-coloured fruit bats (*Eidolon helvum*) in Sub-Saharan Africa. The study location in Ghana is highlighted with the black outline around the country border and the inset box showing the location in Accra. (B) The location of two sampling sites in Accra: the *E. helvum* captive colony (orange diamond) and the wild *E. helvum* population that sourced the bat flies restocked into the captive colony in January 2012. (C) Timeline of the study, including the 14 time points where blood was sampled from captive *E. helvum*, the sampling or transfer of bat flies, and a qualitative description of the bat fly population density over time. Figure 2 long description.

Using a captive colony of straw-coloured fruit bats (*Eidolon helvum*) in Accra, Ghana (Figure 2A, B), the community dynamics of *Bartonella* bacteria were monitored in bats over 3 years (Figure 2C). During this experiment, the presumed vectors (bat flies) declined in density within the colony but were then restocked from a nearby wild source population of *E. helvum* (Figure 2B, C). The experiment thus controls parasite dispersal across 2 scales: the captive colony is closed to immigration (pups enter the colony uninfected), and transmission is manipulated via changes in the bat fly population size. By manipulating parasite dispersal, the effect of among-host dispersal is minimized and the effects of local, within-host effects (ecological drift and species sorting) on parasite dynamics and diversity can be observed. We hypothesized that *Bartonella* communities in the colony would respond to changes in among-host dispersal/transmission by bat flies. Specifically, we predicted that infection prevalence and diversity would at first decline concurrently with the bat fly population and then increase upon restocking of flies, thus providing experimental evidence that bat flies are vectors of *Bartonella* in bats. We hypothesized that limitation of parasite dispersal would result in stochastic losses of rare *Bartonella* species and increases in community beta diversity due to ecological drift and shifts in the rank abundance of *Bartonella* communities over time due to species sorting. Finally, we hypothesized that potential interactions among *Bartonella* species would be detectable based on coinfection frequencies, specifically evidence of competition and/or facilitation. This work expands our understanding of *Bartonella* dynamics in natural communities, particularly in bats and their ectoparasites. More broadly, this experiment deepens our understanding of the processes that affect parasite communities, patterns which may be compared with those seen in communities of free-living or mutualistic organisms.

## Materials and methods

### Study system and experimental design

*Eidolon helvum* (Chiroptera: Pteropodidae) is a long-lived, tree-roosting bat species distributed across Sub-Saharan Africa (Figure 2A) that can form enormous colonies during the local dry season (Hayman et al., 2012; Fahr et al., 2015). The bat flies (*Cyclopodia greefi*; Diptera: Nycteribiidae) hosted by this bat are wingless but can move among hosts within densely populated roosts. At least 6 distinct *Bartonella* species have been previously described in *E. helvum* (Kosoy et al., 2010; Bai et al., 2015) and the same species plus additional variants have been detected in *C. greefi* (Billeter et al., 2012; Kamani et al., 2014; McKee et al., 2024).

Materials for this study come from a captive population of *E. helvum* bats (Figure 2B) in Accra, Ghana (Baker et al., 2014). Briefly, the captive facility is a double-fenced hexagonal 27.5 m diameter and 3.5 m high structure; a solid metal roof and cladding at the base, along with the double-fenced walls, prevent contact with other animals. The captive population was founded by 3 cohorts (Supplementary Table S1) of mixed age and sex (*n* = 78) collected from a large seasonal colony in Accra (Hayman et al., 2012). The cohorts entered the colony in July 2009, November 2009 and January 2010; 2 additional cohorts were born in captivity in April 2010 (produced by mating between wild bats before they entered the colony) and 2011 (produced by mating in captivity). All 13 captive-born neonates were matched to the dam they were attached to at the first sampling point after birth. Ethics approval for bat capture and the fly restocking experiment was obtained from the Zoological Society of London Ethics Committee (WLE/0467), the Veterinary Services Directorate of Ghana, and the Wildlife Division of the Forestry Commission of Ghana.

Bats were assigned to age classes and sex upon entry to the colony and afterward according to approximate birth date and secondary sexual characteristics (Peel et al., 2016): neonate, juvenile, sexually immature adult, and sexually mature adult. Passive-integrated transponder (PIT) tags were implanted in each bat either at entry or shortly after birth to uniquely identify each bat and adult bats additionally received necklaces with alphanumeric codes. Although 112 total bats entered the colony, 25 bats left the colony either through recorded mortality (*n* = 12) or presumed mortality after being recorded missing for ≥ 3 sampling points (*n* = 13). Furthermore, not all bats had complete sample histories throughout the experiment because they intermittently escaped capture for processing.

Blood samples were taken from the captive bats every 2 months in 2009 and 2010 and every 4 months in 2011 (Table S1; see Appendix 1 for sampling protocol). On 6 March 2010 (day 221), a sample of bat flies (*C. greefi; n* = 28) was removed from the colony for testing for the presence of *Bartonella* DNA (Figure 2C). The prevalence of bat flies on bats in the colony was 27% (6/22) in November 2009 and 46% (28/61) in March 2010. The ectoparasite prevalence in the source population for the colony bats was higher when sampled in January 2012, at 78% (39/50). This prevalence was also substantially lower than that observed in wild populations of *E. helvum* on islands in the Gulf of Guinea, where bat fly prevalence ranged from 60% to 92% (McKee et al., 2024). From March 2010 onwards, bat flies were not observed on the colony bats nor were any other ectoparasites (fleas, ticks, mites) recorded, so it is assumed that little to no horizontal vector-borne transmission was occurring. The reason for the decline in bat flies during this period is unknown, but we suspect that the adult bat fly population present at the beginning of the study progressively died off since the estimated longevity of adult nycteribiids is only 3–5 months (Marshall, 1970). The conditions in the colony may have also prevented females from depositing prepupae on the concrete and metal surfaces of the colony housing, or the young flies failed to emerge under these conditions. Thus, the population was not replenished by new births. However, we cannot dismiss the possibility that other exogenous factors (temperature, humidity, or precipitation) may have also contributed to increased adult mortality or decreased births in the bat flies, which has been explored in recent studies (Andrianiaina et al., 2025).

To test the effect of restoring transmission on *Bartonella* community dynamics and to provide evidence that bat flies are vectors, bat flies were experimentally added to the colony. On 17 January 2012 (day 903), a sample of adult bat flies (*N* = 91) was taken from the original wild source colony of bats (Figure 2C). From this total, 73 were randomly assigned to approximately half of the bats in the colony (*N* = 38 bats, 1–4 flies per bat). The remaining 18 bat flies and blood samples from bats in the wild source colony (*N* = 50) were used as comparison groups for the captive colony regarding *Bartonella* prevalence and diversity. Blood samples from captive bats were subsequently taken at 3 additional time points after the addition of flies: 31 January 2012, 14 February 2012 and 15 March 2012. In total, 910 blood samples were taken from the captive colony over 14 time points from 2009 to 2012 (a period of 961 days), of which 905 samples could be definitively assigned to an individual by PIT tag or necklace ID. An additional 50 blood samples and 18 flies were taken from wild bats on 17 January 2012.

### Bacterial detection and gene sequencing

The focus of this study was on changes in *Bartonella* infection prevalence and the relative frequency of different *Bartonella* species in bats, so a molecular detection and sequencing approach capable of distinguishing coinfecting species was used. Bat blood and fly samples were tested for the presence of *Bartonella* DNA using a multi-locus PCR platform (Bai et al., 2016) targeting fragments of the 16S–23S ribosomal RNA intergenic spacer region (ITS), citrate synthase gene (*gltA*) and cell division protein gene (*ftsZ*). Each of these loci is capable of distinguishing among *Bartonella* species and subspecies (La Scola et al., 2003), but may have amplification biases toward different *Bartonella* species in a sample (Kosoy et al., 2018; Himsworth et al., 2020). Thus, the purpose of this multi-locus approach was to confirm the detection of *Bartonella* DNA and to indicate across loci whether infections with multiple species were present. Further quantification of *Bartonella* infection load was performed using real-time PCR targeting the transfer-messenger RNA (*ssrA*). Sequences were verified as *Bartonella* spp. using the Basic Local Alignment Search Tool (BLAST; https://blast.ncbi.nlm.nih.gov/Blast.cgi). Samples were only considered positive if a significant match was observed, even if there was a positive real-time PCR result (cycle threshold value [Ct] < 40). *Bartonella* sequences with multiple peaks in the electropherogram were separated into 2 or more distinct sequences by comparison with previously obtained *Bartonella* sequences from *E. helvum* and *C. greefi* (Billeter et al., 2012; Bai et al., 2015); this is possible due to the genetic dissimilarity of the *Bartonella* species found in these hosts and the distinct patterns of substitutions found in the sequenced markers (Bai et al., 2015; McKee et al., 2024). Due to the frequency of multiple sequences obtained from these loci, conflicting sequences across genes were interpreted as evidence of coinfection rather than homologous recombination, and thus we report counts of sequences from distinct *Bartonella* species within a sample as recommended by Kosoy et al. (2018). All variants of *Bartonella* sequences sharing < 95% sequence similarity with previously identified *Bartonella* species were submitted to GenBank. PCR primers and protocols are listed in Tables S2 & S3. Additional details on bacterial detection and phylogenetic analysis are provided in Appendix 1.

### Data recording and statistical analyses

Relevant measures of *Bartonella* infection prevalence, infection load, and diversity were recorded or calculated to assess changes that occurred during the experiment, including before and after the addition of bat flies to the captive colony. *Bartonella* infection prevalence within the captive bat colony, in sampled wild and captive flies, and from wild bats was reported based on the number of tested bats or flies that were positive at one or more loci (ITS, *gltA, ftsZ, ssrA*). Wilson scores were used to calculate 95% confidence intervals for single infection and coinfection prevalence. *Bartonella* alpha diversity was measured by *Bartonella* species richness and Shannon number, i.e., the effective number of species or the exponent of Shannon’s diversity index (Jost, 2006); species richness within each sample based on the number of loci with positive sequences was also recorded. The frequency of *Bartonella* species detected in the colony was calculated from the presence/absence of each *Bartonella* species in the total number of sequences obtained across all loci for a given sample, including separate sequences obtained from the same locus. A custom bootstrapping procedure with 1000 samples from the observed multinomial distribution of *Bartonella* species frequencies was used to estimate 95% confidence intervals around measures of alpha diversity. *Bartonella* beta diversity was calculated across sampled bats and flies based on species presence/absence using the Jaccard index within the *vegdist* function in the R package *vegan* (Oksanen et al., 2025; R Core Team, 2025). Infection load was recorded as the real-time PCR Ct value for each sample. Additionally, for each bat, the time until becoming infected after first entering the colony and the duration of infection for the most persistent *Bartonella* species were recorded. These measures help to track whether certain demographic groups are more affected by the addition of flies and compare changes in the relative frequency of *Bartonella* species over time, respectively. Change points in *Bartonella* prevalence, infection load, and diversity measures were detected with segmented regression using the R *segmented* package (Muggeo, 2003, 2024). Binomial generalized regression models (GLM) were fit to compare changes in infection status for bats that did or did not receive bat flies in January 2012, including age and sex as potential confounding variables. Mean infection durations for *Bartonella* species were estimated using a Bayesian approach by fitting lognormal distributions to the data with Stan using the *rstanarm* package (Carpenter et al., 2017; Gabry et al., 2025). Multinomial and binomial likelihood ratio (LR) tests adapted from Pepin et al. (2013) were performed to find statistical associations between coinfecting *Bartonella* species and to detect changes in the relative frequency of *Bartonella* species during the study period. For additional details regarding regression analyses, Bayesian models, and likelihood ratio tests, see Appendix 1.

## Results

### Phylogenetic analysis of detected bacteria

*Bartonella* infections in bats and bat flies were identified as 6 previously characterized species based on ITS, *gltA* and *ftsZ* sequences: *Bartonella* spp. E1–E5 and Ew (Kosoy et al., 2010; Bai et al., 2015). Two additional genogroups identified by *gltA* sequences, *Bartonella* spp. Eh6 and Eh7 (Figure S1), were similar to sequences previously obtained from *C. greefi* collected from *E. helvum* in Ghana and islands in the Gulf of Guinea (Billeter et al., 2012; McKee et al., 2024). Sequences identified as Eh6 and Eh7 were also detected among *ftsZ* and ITS sequences (Figures S2 & S3). Phylogenetic analysis of concatenated *ftsZ* and *gltA* sequences distinguished Eh6 and Eh7 from other *Bartonella* species associated with *E. helvum* or other bat species (Figure S4). See Appendix 2 for more details on phylogenetic analysis.

### *Bartonella* infection prevalence and effects of bat fly restocking

**Figure 3.**
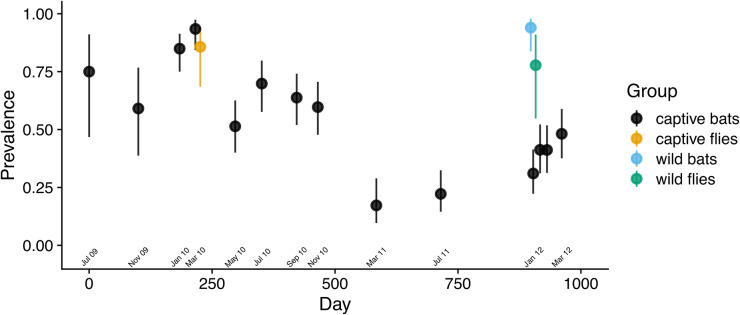
*Bartonella* infection prevalence in a captive colony of *E. Helvum* over time. Bats and bat flies were considered positive if a *Bartonella* sequence was obtained from one or more genetic markers. Wilson score 95% confidence intervals were drawn around prevalence estimates at each sampling time point. Figure 3 long description.

As predicted, *Bartonella* prevalence in the captive colony changed with the population density of bat flies. *Bartonella* prevalence in the first 3 cohorts was high at colony entry, then declined concurrently with the observed decline in the bat fly population (Figure 3). After flies were restocked, prevalence increased from 31% on day 903 to 48% on day 961. This change was reflected in the segmented regression analysis (Figure S6A; Table S7) with a shift from positive to negative slope near March 2010 (day 221) and a shift from negative to positive slope around January 2012 (day 903). The trend in *Bartonella* prevalence in the colony over time was similar if bats were considered positive for *Bartonella* with a threshold of at least 1, 2, 3, or all genetic markers being positive (Figure S7).

The effect of bat fly restocking on *Bartonella* prevalence was more pronounced for some age classes of bats than others (Figure S8A). All sexually immature bats were infected at entry and by the end of the study, but there was an increase in the proportion of adult bats infected by the end of the study compared to the start, from 81% to 94%. Bats born into the colony in 2010 and 2011 were *Bartonella*-negative at their first sampling event. By the end of the experiment, 88% of these bats had become infected (Figure S8A). Using data from the 112 bats that were tested for *Bartonella* infection more than once during the captive study, a binomial GLM was fit to test the effects of age and sex on whether bats became *Bartonella*-positive during the experiment. The largest effect was observed in neonates/juveniles born into the colony (Table S4): compared to adults, neonates/juveniles were significantly more likely to become positive by the end of the study (log odds ratio = 3.9, z = 5.8, *P* < 0.001). There was no significant effect of sex on the change in *Bartonella* infection for any age class (Table S4).

To further examine the effect of restocking flies on 17 January 2012, we fit logistic GLMs for 2 additional outcomes: (1) whether bats were *Bartonella*-positive at any time point after flies were restocked on 17 January 2012, and (2) whether bats became positive or bats that were already positive changed *Bartonella* species after fly restocking. In both models, we used data on the 84 bats that were present in the colony on 17 January 2012 and subsequent time points. Both models included age class, sex, whether bats received flies on 17 January 2012, and infection status prior to 17 January 2012 as predictors. The first model identified a significant effect of age on *Bartonella* infection status showing that neonate/juvenile bats were significantly more likely to be *Bartonella*-positive after flies were restocked on 17 January 2012 compared to mature adults (log odds ratio = 1.69, *z* = 2.62, *P* = 0.024; Table S5A, B). There was no significant effect of prior infection or sex on this outcome (Table S5A), and while receiving flies did increase the likelihood of infection after 17 January 2012, this effect was also not statistically significant (*z* = 1.61, *P* = 0.11). Based on the second model, age class was also an important predictor of whether a bat became positive or changed *Bartonella* species after flies were restocked (Table S5C, D). Like the first model, the effects of sex and prior infection were not significant (Table S5C). However, the effect of flies was statistically significant in this model (*z* = 2.0, *P* = 0.045). This suggests that after adjusting for age class, sex, and prior infection status, bats that received flies on 17 January 2012 were more likely to become newly infected with *Bartonella* or change *Bartonella* species.

Bat fly restocking had similar effects on measures of infection load in the colony. Infection load in each sample, as measured by RT-PCR cycle threshold (Ct) values (Figure S5), reached a peak in March 2010, then declined before sharply increasing after the restocking of flies. This trend is reflected in the segmented regression of this measure, with a shift from positive to negative slope near day 221 and a shift from negative to positive slope near day 903 (Figure S6B; Table S7). Coinfection prevalence showed a peak near January 2010 (day 184) and declined until July 2011 (day 715) when it began to increase again (Figure S9). However, only the shift in slope for coinfection prevalence near July 2011 was statistically significant (Figure S6C; Table S7). These data show that multiple measures of *Bartonella* infection in the colony changed over time, particularly in response to the restocking of flies in January 2012. For additional details on prevalence and load in bat flies and wild bats collected in March 2010 and January 2012, see Appendix 2.

### Patterns of *Bartonella* diversity

Similar to infection prevalence and load, *Bartonella* diversity measures changed in response to bat fly population density. *Bartonella* diversity was measured at 2 scales, at the colony level and at the individual host level. *Bartonella* species richness and evenness (Shannon index) measured colony-level alpha diversity. The number of *Bartonella* species in an individual sample and beta diversity (Jaccard index) measured individual-level diversity. Diversity measures showed qualitatively similar patterns during the early phase of the experiment (Figures S10 & S11): an initial increase with the entry of the first 3 cohorts into the colony, reaching a maximum in January 2010, followed by a decline. Diversity measures increased again until the restocking of flies in January 2012 and then declined slightly (or remained flat in the case of species richness). The observed trends were only partially reflected by segmented regression breakpoints. Segmented regression detected only one breakpoint each in the timelines for species richness, species evenness, and the number of *Bartonella* species in an individual sample (Table S7). A shift from positive to negative slope was detected in January 2010 for species richness (Figure S12A), whereas a change from negative to positive slope was detected for species evenness and the number of species in an individual sample between November 2010 and March 2011 (Figure S12B, C; Figure S13A). There were 2 significant breakpoints detected in the timeline of beta diversity, changing from negative to positive slope in May 2010 and from positive to negative slope in January 2012 (Figure S13B; Table S7). For details on diversity measures in bat flies and wild bats collected in March 2010 and January 2012, see Appendix 2.

### Shift in *Bartonella* species frequencies

**Figure 4.**
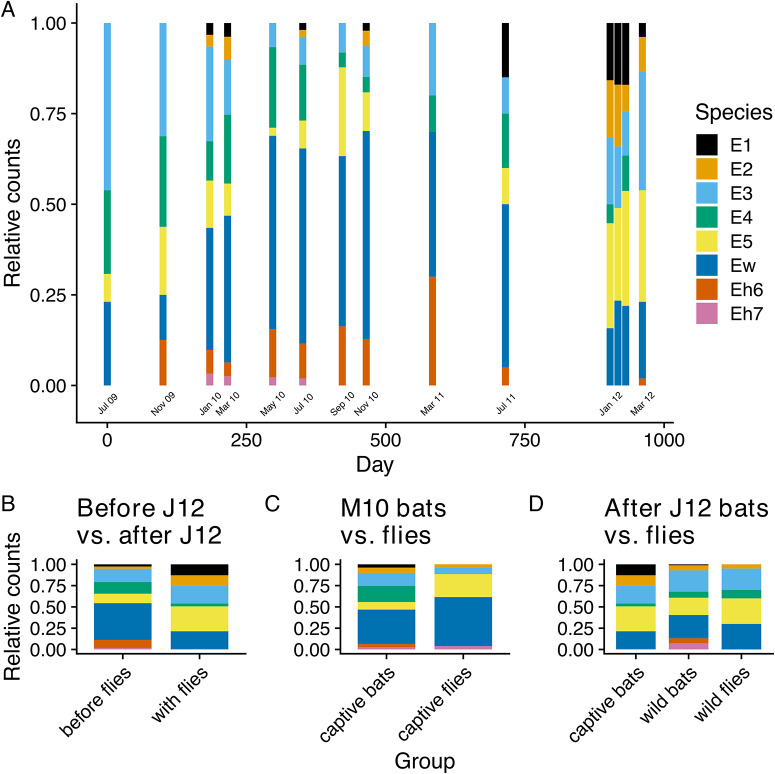
Changes in the relative counts of *Bartonella* species in the captive colony over time (A–B) and between sampled bat flies and their respective bat populations (C–D). Relative counts (A) at each time point were estimated from the presence/absence of *Bartonella* species based on any positive sequence from ITS, *gltA* and *ftsZ*. For panels A and B, the month labelled in bold font on the *x*-axis shows when bat flies were added to the bat colony. Tests for differences in the relative counts of species were performed between bats in the captive colony before and after bat flies were restocked on 17 January 2012 (B); between bat flies sampled from the colony and the captive bat population in March 2010 (C); and between bat flies and wild bats sampled in January 2012 and the captive colony population after flies were added (D). Figure 4 long description.

*Bartonella* species observed in the colony varied in their frequencies with an apparent shift in the dominant species during the study (Figure 4A). While rarer species E1, E2 and Eh7 were not observed at all time points, E1 and E2 were consistently observed over the duration of the study. In contrast, the rarest species Eh7 was not observed after July 2010, even after flies were added to the colony. Species Eh6 was also uncommonly observed during the study, went unobserved for 3 time points in 2012, but was observed again in March 2012.

**Figure 5.**
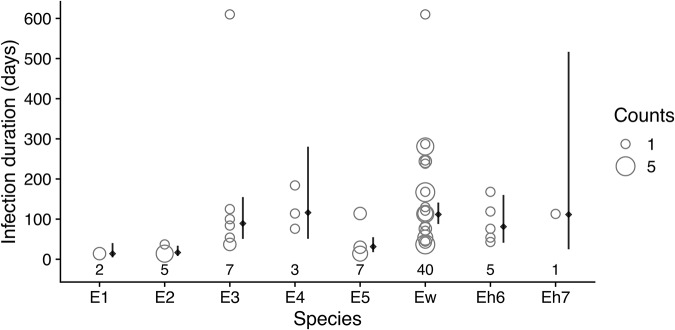
Duration of *Bartonella* species infections in serially infected individuals. For each *Bartonella* species, the numbers below the points are counts of individual bats that had the *Bartonella* species as their longest-lasting infection (i.e., the *Bartonella* species was present for the most sequential time points). The infection durations in days for all serially infected bats are plotted as open circles with the width proportional to the number of individuals with the same infection duration. Solid diamonds and lines to the right of points show the estimated mean duration and 95% posterior intervals using Bayesian lognormal regression. Figure 5 long description.

As noted above, beta diversity decreased after May 2010 when the bat fly population was decreasing, reached another maximum in January 2012, and then decreased again after flies were restocked (Figure S11). These decreases in beta diversity correspond with periods of expansion by some species within the colony that appear to homogenize beta diversity. During the period from January 2010 to July 2011, Ew became the most abundant species in the colony (Figure 4A). Another measure of this species’ dominance in the colony is the duration of its infections in individual bats. For each bat that was sampled more than once and was recorded as having the same *Bartonella* species for a sequential period, we tabulated which species was present for the most time points (Figure 5) and estimated the mean duration of infection using Bayesian regression (Table S6). Among *Bartonella* species, Ew was the longest-lasting infection in the highest number of bats (*n* = 40), with an estimated mean of 112 days (95% posterior interval: 88–141 days). Species E4 and Eh7 were similarly long-lasting infections but were observed in relatively fewer bats (*N* = 3 and 1, respectively; Table S6).

Beginning around March 2011, the frequency of Ew began declining while species E1, E2, and E5 increased (Figure 4A). Dividing the study into 2 parts – before flies were restocked (July 2009 to July 2011) and after flies were added (January 2012 and after) – a clear difference in the relative frequency of *Bartonella* species was observed (Figure 4B). This shift in frequency after the addition of flies was significant according to a multinomial LR test (D = 183.3, df = 7, *P* < 0.001) and individual binomial LR tests for all species, except for E3 (Table S8). Significant differences were also observed in the frequencies between bat flies and sampled bat populations in March 2010 and January 2012 (Figure 4C, D; Table S9). These data, along with the changes in alpha and beta diversity measures, demonstrate that *Bartonella* communities shifted substantially over time, coinciding with the changes in the bat fly population density.

### Interactions between *Bartonella* species

**Figure 6.**
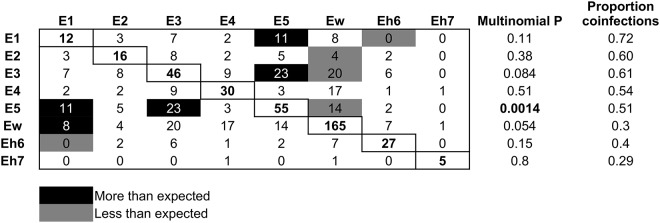
Patterns of *Bartonella* species coinfection. Rows are the focal species and columns are the partner infections. Numbers in the boxes are counts of coinfections between each pair of species; single infection counts for each species are on the diagonal. Black boxes show coinfections that occurred more frequently than expected, grey boxes show those that occurred less frequently than expected and white boxes show those with no significant pattern. Expected counts were based on the frequency of single and double infections of each *Bartonella* species, and significance was based on multinomial and binomial tests. The proportion of infections by each *Bartonella* species that were also coinfections is shown in the last column. Figure 6 long description.

Using multinomial and binomial LR tests on coinfection frequencies, there was evidence of both negative and positive interactions between *Bartonella* species over the period of the experiment (Figure 6). Bats infected with Ew were significantly less likely to be coinfected with E2, E3 and E5; a reciprocal negative effect on Ew from these species was not detected. Related to this, the proportion of Ew infections that were also coinfections was low (30%), in contrast to its high frequency in the population over time (Figure 4A). Species E1 and Eh6 had a reciprocal negative effect on each other. Reciprocal positive effects (i.e., more coinfections than expected) were found between species E3 and E5 and between species E1 and E5. Also, bats were more likely to be coinfected with Ew if they were already infected with E1, but there was no significant reciprocal effect of Ew on E1 (Figure 6).

## Discussion

Parasites do not infect hosts in isolation but instead form diverse communities within hosts that vary over time. However, it is unclear how the same forces that affect diversity in communities of free-living, sexually reproducing organisms act in the same way or with different strengths in parasite communities (Sutherland et al., 2013). Furthermore, there are few studies that have tracked ecological communities of parasites over time to examine the forces shaping diversity (Vidal-Martínez and Poulin, 2003; Fallon et al., 2004; Cohen et al., 2015; Budischak et al., 2016; Moss et al., 2020). This study tested how well predictions of community ecology theory (Vellend, 2010) apply to host–vector–parasite systems through an approach that manipulated parasite dispersal among hosts within the population by capitalizing on a natural change in the population density of the putative vector. Restriction of parasite dispersal minimized the effect of among-host transmission on *Bartonella* communities within individual hosts, thereby allowing the possible effects of ecological drift and species sorting on parasite community diversity to be measured. At the same time, observed trends in the prevalence and diversity of *Bartonella* infections within the colony over the course of vector population decline and restocking indicate that bat flies are biological vectors of *Bartonella* in bats. Overall, the experiment shows that *Bartonella* communities are affected by dispersal, drift, and ecological selection in similar ways to free-living organisms, although numerous forms of ecological selection might be acting simultaneously.

We first hypothesized that *Bartonella* communities in the colony would respond to changes in among-host dispersal/transmission by bat flies. Specifically, we predicted that infection prevalence and diversity would decline concurrently with the bat fly population and then increase upon restocking of flies. The results indicate that *Bartonella* prevalence and infection load declined along with the bat fly population, then increased when flies were added in January 2012. This effect was seen across the whole population but had a stronger effect on young bats born in the colony, likely attributable to their lack of prior exposure to *Bartonella* while flies were in low density. Only a few vectors of *Bartonella* bacteria have been confirmed through controlled exposure of hosts to infected vectors (Tsai et al., 2011; Morick et al., 2013), and all of these have been in non-bat hosts. A previous study by Jardine et al. (2006), demonstrated declines in *Bartonella* prevalence after an experimental insecticide treatment reduced flea densities on Richardson’s ground squirrels (*Spermophilus richardsonii*), indicating that fleas are important vectors of *Bartonella*. The role of bat flies as *Bartonella* vectors had been speculated in previous work on *Bartonella* dynamics in bats (Morse et al., 2012; Brook et al., 2015; Stuckey et al., 2017; Sándor et al., 2018; Nabeshima et al., 2020; Fagre et al., 2023), but our experimental study provides conclusive support for nycteribiid transmission of *Bartonella* in bat hosts.

Multiple measures of *Bartonella* diversity decreased over the corresponding period when flies were at low density. This decline may be attributed to the stochastic loss of rare species and the increase in relative frequency of some species, specifically Ew, through persistent infection. Bartonellae are known to cause persistent infections in their reservoir hosts, with frequently relapsing bacteraemia that can occasionally go dormant and reactivate through the clonal expansion of antigenic or phase variants, or potentially coinfecting *Bartonella* species (Kosoy et al., 2004b; a; Harms and Dehio, 2012; Pulliainen and Dehio, 2012). Interestingly, all diversity measures increased before the restocking of flies, reaching a local peak in diversity in January 2012 before declining again. This second decline could be attributed to the decline of the dominant Ew, allowing potentially latent infections by other species (E1, E2, E3, E5) to emerge as the dominant species infecting the bat population. The dominance of these species in the colony continued after flies were restocked and among-host transmission was restored, thus causing a short decline in diversity measures. These patterns indicate that dispersal of infections by flies is not only key to maintaining infection prevalence, but also the long-term maintenance of *Bartonella* community diversity in bats.

We also hypothesized that limitation of parasite dispersal would result in stochastic losses of rare *Bartonella* species and changes in community beta diversity via ecological drift and shifts in the rank abundance of *Bartonella* communities due to species sorting. The rarest species in the community, *Bartonella* species Eh7, was lost during the study and was not restored, even when flies were restocked. This failure was likely due to a sampling effect, wherein flies carry only a subset of *Bartonella* species, therefore limiting opportunities for effective reintroduction of rare species. This is especially true for this experiment given the small number of flies (N = 73) added to the colony. As noted above, beta diversity did not exhibit the expected increase when the fly population declined. Instead, there was a decrease in beta diversity due to the dominance of species Ew. This dominance of Ew was the most conspicuous trend in the dynamics of the *Bartonella* community over most of the study, except for at the end of the experiment when there was a shift towards the next most abundant species, E5, and other lower-ranked species. This shift towards E5 and the decline in Ew occurred before the addition of flies and was independent of the effects of among-host dispersal (due to the low density of flies at this time). We speculate that this is an emergent pattern due to within-host selection against Ew by the host immune system. Specifically, as Ew came to dominate within the population and in individual bats, it may have become the primary target of host immune responses. As Ew was eliminated, this allowed for the emergence of other, latent infections within coinfected bats. Thus, without dispersal of *Bartonella* species by bat fly vectors, we hypothesize that ecological drift and species sorting by the host immune system cause observable changes in bacterial communities.

Finally, we hypothesized that interactions among *Bartonella* species would be detectable based on coinfection frequencies, providing evidence of competition or facilitation in pathogen communities. While most interactions were not significant, species Ew has negative effects on several species and is typically present with few coinfections. In contrast, positive effects were observed between species E1, E3 and E5, which show a much higher frequency of coinfection. These results indicate that parasitic bacteria like *Bartonella* do have measurable ecological interactions that are not uniformly competitive. These positive interactions could have played a role in the replacement of Ew with E5 and other species late in the study.

From just a single experiment, we can make several inferences about the ecology of *Bartonella* infections in bats. First, they can be persistent, lasting potentially hundreds of days. Other studies have alluded to the possibility of persistent *Bartonella* infection with periodic recrudescence in rodents (Kosoy et al., 2004b; a; Bai et al., 2011; Goodrich et al., 2020) and bats (Becker et al., 2018); however, these studies were conducted in open populations where reinfection by vectors was likely frequent. Although we cannot rule out that some reinfection occurred due to a small, remnant bat fly population in the colony, the decline in bat fly density to an undetectable level should have reduced reinfections relative to studies of wild populations. Second, *Bartonella* community diversity can be driven by dispersal, drift, and ecological selection (i.e., species sorting), as predicted by ecological theory (Vellend, 2010; Seabloom et al., 2015). The current study has shown that when dispersal is limited, the effects of ecological drift and selection can be more apparent. Two types of ecological selection can occur in these parasite populations, either through interactions with the host immune system or through interspecific interactions. As noted above, the immune system may lead to periodic selection against the dominant infecting species, a negative frequency-dependent mechanism that might help maintain diverse parasite communities (Fallon et al., 2004; Christie and McNickle, 2023).

Dominance appears to be a similar facet of the composition of pathogen communities (de Jong et al., 2025; Pinotti et al., 2019) as it is in free-living organisms (Smith and Knapp, 2003). The dominance of Ew may thus stem from multiple facets of its ecology. First, it appears to be persistent within bats (Figure 5), and second, it appears to be readily taken up by flies (Figure 4C, D). We note that Ew is also the most clonal, i.e., genetically homogenous, species in the community and might be a more recently evolved or introduced species in *E. helvum* (Bai et al., 2015). While there was no evidence that Ew caused higher infection loads (by Ct value), the resolution of our sampling protocol probably was not high enough to detect this.

This study has several limitations that could be addressed with additional studies of this system. Firstly, the decline and restocking of bat flies in the colony was not precisely controlled, nor replicated in multiple bat populations. While this natural experiment provides promising data identifying the role of bat flies as *Bartonella* vectors, additional field studies are needed to verify their competence while controlling for other environmental factors that may affect transmission. Such experiments might involve controlled exposure of *Bartonella*-negative bats and confirmation of the exposure route. Alternative routes might include bat fly bite, requiring tropism of the bacteria to the salivary glands, or contamination through bat fly faeces, requiring replication in the fly gut and persistent shedding of viable bacteria in faeces.

We also recognize that the molecular methods we used to detect *Bartonella* infections cannot give us a full picture of the microbial community dynamics occurring in this system. For instance, our PCR-based approach almost certainly underestimated the frequency of coinfections in bats. Multiplexed high-throughput sequencing on blood samples could detect coinfections with higher sensitivity and provide better measurements of the relative abundance of *Bartonella* species within the bat hosts (Power et al., 2021; Bai et al., 2023). Isolation of *Bartonella* cultures could provide opportunities to inspect growth curves throughout the infection cycle to see if some species have any growth advantages (Lynch et al., 2011; Gutiérrez et al., 2017). Finally, whole-genome sequencing from blood or cultures could identify genetic changes in *Bartonella* in response to host immune selection (Rodríguez-Pastor et al., 2024). Additional studies could try other methods to examine immune function in bats (Boughton et al., 2011) in response to *Bartonella* infection to confirm the existence of frequency-dependent selection against *Bartonella* species and to help determine the appropriate epidemiological models to explain *Bartonella* infection dynamics (Brook et al., 2017). Other forms of interference or resource competition must be explored further (Pedersen and Fenton, 2007), perhaps through controlled infection experiments.

In summary, this study has contributed to a more comprehensive understanding of the ecology of *Bartonella* species in bats and connects with broader community ecology theory developed in free-living and symbiotic organisms (Vellend, 2010; Costello et al., 2012; Miller et al., 2018). In this experiment, limitation of dispersal led to declines in local *Bartonella* species diversity in individual bats, a pattern that fits well with predictions from patch dynamics or mass effects models of metacommunities (Leibold et al., 2004). The results also show that not all bacterial interactions are negative, even those that presumably share the same niche. This parallels the recognized importance of positive species interactions in plant communities (Bertness and Callaway, 1994) and among bacterial taxa in animal microbiomes and aquatic habitats (Faust et al., 2012; Ju and Zhang, 2015; Hegde et al., 2018). A recent study by Gutiérrez *et al.* (Gutiérrez et al., 2018) on *Bartonella* infections in desert rodents showed a mixture of negative, neutral and positive interactions similar to the present study. Theoretical and experimental studies suggest that communities remain stable through a predominance of neutral or weak species interactions that can attenuate large competitive or facultative effects (McCann, 2000; Aschehoug and Callaway, 2015). Weak interactions, paired with the frequency-dependent selection discussed above, could provide a model for understanding how *Bartonella* species and other parasitic microorganisms coexist in communities within their hosts. Such mechanisms could allow bacteria to share a niche or split it temporally, which could lead to periodic shifts in the dominant species but maintain the community as a whole. Future work using this system and similar longitudinal studies on other pathogens in natural host populations could lead to additional insights into the nature of microbial communities and the broad ecological processes that act across taxonomic and spatial scales.

## Supporting information

10.1017/S0031182026101656.sm001 McKee et al. supplementary material 1 McKee et al. supplementary material

10.1017/S0031182026101656.sm002 McKee et al. supplementary material 2 McKee et al. supplementary material

## Data Availability

The data that support the findings of this study are available in the supplementary material of this article. Representative sequences for *Bartonella* genogroups Eh6 and Eh7 from *E. helvum* and *C. greefi* have been submitted to GenBank under the accession numbers MN249715–MN249720 and MN250730–MN250788. Phylogenetic trees, R code and additional data sheets are available on GitHub (https://github.com/clifmckee/eidolon_captive_bartonella).

## Supplementary material

The supplementary material for this article can be found at https://doi.org/10.1017/S0031182026101656.

## Figure 1 Long description

The diagram illustrates parasite metacommunity dynamics within host populations. Two dashed circles represent host populations, each containing colored dots symbolizing different parasite species. The left circle includes orange and green dots, with labels 'Speciation', 'Selection' and 'Drift' positioned near the orange dot. Arrows labeled 'Parasite dispersal (transmission)' connect the two circles, indicating movement of parasites between host populations. The right circle also contains orange and green dots, showing the interaction and transmission of parasite species between hosts.

Navigate back to Figure 1.

## Figure 2 Long description

The image contains three panels labeled A, B and C. Panel A is a map with the horizontal axis labeled Longitude, ranging from negative 20 to 60 and the vertical axis labeled Latitude, ranging from negative 40 to 40. The map shows a shaded geographic range across Sub-Saharan Africa. A small outlined region marks the location of Ghana on the map. Panel B is a detailed map of Accra with the horizontal axis labeled Longitude, ranging from approximately negative 0.30 to negative 0.10 and the vertical axis labeled Latitude, ranging from approximately 5.50 to 5.65. Two symbols are plotted: a diamond shape marking the captive colony site at approximately negative 0.20 longitude and 5.60 latitude and a triangle shape marking the wild population site at approximately negative 0.15 longitude and 5.55 latitude. Panel C is a timeline with the horizontal axis showing dates from 28 July 2009 to 14 May 2012. Two horizontal tracks are labeled Treatment and Timeline. The legend for bat fly sampling states: diamond shape means flies sampled from captive colony and triangle shape means flies transferred from wild bats to colony. The legend for status of bat flies states: a thin line means entered with cohorts, a medium line means low density and a thick line means restocked. On the Treatment track, a diamond shape appears at approximately February 2010 and a triangle shape appears at approximately February 2012. On the Timeline track, open circle markers appear at approximately 14 time points spanning from July 2009 to May 2012. The line along the Timeline track transitions between thin segments indicating entered with cohorts, a medium segment indicating low density and a thick segment indicating restocked, with the restocked segment appearing near the end of the timeline around January to May 2012.

Navigate back to Figure 2.

## Figure 3 Long description

A scatter plot with the vertical axis labeled Prevalence and tick labels 0.00, 0.25, 0.50, 0.75 and 1.00. The horizontal axis is labeled Day, with tick labels 0, 250, 500, 750 and 1000. A legend titled Group lists four categories: captive flies, captive bats, wild bats and wild flies. Points are plotted at multiple day values, with vertical error bars on each point. Several points appear between day 0 and day 500 at prevalence values around 0.50 to 0.90. Additional points appear between about day 600 and day 800 at prevalence values around 0.15 to 0.30. A cluster of points appears near day 950 to day 1000 at prevalence values around 0.30 to 0.50 and two higher points near the same day range appear around 0.80 and around 0.95.

Navigate back to Figure 3.

## Figure 4 Long description

The image contains four stacked bar graphs. The first graph (A) shows relative counts of species over time, with days labeled approximately from 0 to 1000. Species E1 and E2 are prominent on most days, with notable shifts in species dominance. The second graph (B) compares relative counts before and after January 2012, showing a change in species composition with flies. The third graph (C) compares captive bats and captive flies, highlighting differences in species distribution, with E1 being more prevalent in captive flies. The fourth graph (D) compares captive bats, wild bats and wild flies after January 2012, showing variations in species presence, with E1 and E2 being dominant in wild flies. Each graph uses a vertical layout with stacked bars representing species E1, E2, E3, E4, Ew, Eh6 and Eh7. The y-axis for all graphs is labeled Relative counts, ranging from 0.00 to 1.00, indicating proportions of species presence.

Navigate back to Figure 4.

## Figure 5 Long description

The horizontal axis shows species categories labeled E1, E2, E3, E4, Ew, Eh6 and Eh7. The vertical axis label is Infection duration (days), with tick labels from 0 to 600. A size legend at the right is labeled Counts, with example open circles labeled 1 and 5. Open-circle points are plotted above each species label. Numbers printed below the species labels are 2 under E1, 4 under E2, 3 under E3, 4 under E4, 40 under Ew, 5 under Eh6 and 1 under Eh7. E1 has points near 0 and around 50. E2 has points near 0 and around 50. E3 has multiple points from near 0 up to around 150 and one point near 600. E4 has points around 100 to 200 and one point around 300. Ew has many points clustered between about 50 and 150, with additional points around 200 to 300. Eh6 has points around 50 to 150. Eh7 has a point around 100 and a vertical interval marker extending upward to around 500, with a diamond marker near 100.

Navigate back to Figure 5.

## Figure 6 Long description

The table has columns: E1, E2, E3, E4, E5, Ew, Eh6, Eh7, Multinomial P, Proportion coinfections. Row 1: E1 12, 3, 7, 2, 11, 8, 0, 0, 0.11, 0.72. Row 2: E2 3, 16, 8, 3, 5, 2, 0, 0, 0.38, 0.58. Row 3: E3 7, 8, 46, 9, 23, 4, 0, 0, 0.084, 0.61. Row 4: E4 2, 3, 9, 30, 11, 3, 0, 0, 0.51, 0.54. Row 5: E5 11, 5, 23, 11, 55, 14, 1, 0, 0.0014, 0.51. Row 6: Ew 8, 2, 4, 3, 14, 165, 1, 0, 0.054, 0.3. Row 7: Eh6 0, 0, 0, 0, 1, 2, 27, 0, 0.15, 0.4. Row 8: Eh7 0, 0, 0, 0, 0, 0, 0, 5, 0.8, 0.29. Black boxes indicate more than expected coinfections, grey boxes indicate less than expected.

Navigate back to Figure 6.
