# Supplementary material for: Manipulating vector transmission reveals local processes in Bartonella communities of bats
Source: Parasitology. 2026 Feb 2;153(3):420–31. doi: 10.1017/S0031182026101656 (PMC13215733; doi:10.1017/S0031182026101656)
Supplement: McKee et al. supplementary material 1 — McKee et al. supplementary material [file S0031182026101656sup001.docx]

**Supplemental Information for:**

**Manipulating vector transmission reveals local processes in *Bartonella* communities of bats**

Clifton D. McKee, Colleen T. Webb, Michael Y. Kosoy, Richard Suu-Ire, Yaa Ntiamoa-Baidu, Andrew A. Cunningham, James L. N. Wood, David T. S. Hayman

**Appendix 1. Supplementary methods**

*Sampling protocol*

Captive bats (Table S1) were collected using hand nets after cordoning bats into one quarter of the cage with a curtain system and then placed into a smaller cage until processing. Wild bats (the first three captive cohorts and those from 31 January 2012) were captured from roosts using 6–18 m mist nets or hand nets, then placed in individual cloth bags until processing. While under manual restraint, the surface of the inner wing along the propatagial vein was wiped with 70% ethanol, then 0.2–1.0 ml of whole blood was collected using a citrated 1 ml syringe and transferred into labelled microcentrifuge tubes. After bleeding had ceased, bats were released either into the main area of the enclosure or back to the wild roost. On two occasions, bat flies were collected from bats, March 2010 from the captive colony and January 2012 from the wild source colony. Bat flies were collected from the pelage of bats and placed in individual sterile tubes labelled with the ID number of the host bat. Whole blood was immediately frozen at -80 ºC, or blood clots were separated from serum, then frozen at -80 ºC; bat flies were kept frozen at -80 ºC. All samples were shipped to the Centers for Disease Control and Prevention Division of Vector-Borne Diseases in Fort Collins, CO, on dry ice where they were kept at -20 ºC or below until DNA extraction.

*DNA extraction*

Bat flies were rinsed in 70% ethanol, then in sterile 1x PBS (0.15 M, pH 7.5, CDC, Atlanta, GA) before being transferred to a clean microcentrifuge tube and triturated in 500 μl brain heart infusion (BHI) broth (CDC, Atlanta, GA) using a sterile pestle. Genomic DNA was extracted from bat blood samples and triturated bat flies either by hand using a QIAamp DNA Mini Kit (Qiagen, Valencia, CA) or with a QIAxtractor automated instrument (Qiagen) following the manufacturer’s protocols for tissues (flies) or blood. Extraction controls (blank wells) were included to ensure no cross-contamination occurred during extraction. Extracted DNA was stored in clean microcentrifuge tubes at -20 °C or 4 °C during the duration of pathogen testing.

*Bacterial detection and sequencing*

*Bartonella* spp. were detected via conventional PCR targeting the 16S–23S intergenic spacer region (ITS) via single-step PCR (Diniz *et al.*, 2007) and the citrate synthase (*gltA*) and cell division protein (*ftsZ*) genes via nested PCR (Bai *et al.*, 2016). Quantification of *Bartonella* infection load was performed using real-time PCR targeting the *Bartonella* transfer-messenger RNA gene (*ssrA*) (Diaz *et al.*, 2012). Primers and thermocycler protocols for all real-time and conventional PCR are listed in Tables S2 & S3.

All PCR amplifications were run in a C1000 Touch Thermal Cycler (Bio-Rad, Hercules, CA) with the addition of a CFX96 Real-Time System (Bio-Rad) for real-time PCRs. For all PCRs, positive (*Bartonella doshiae*) and negative (RNase-free water only) controls were included to determine correctly sized amplicons and to detect potential cross-contamination, respectively. PCR products were inspected for the presence of positive amplicons of the correct size by gel electrophoresis using 1.5% agar and GelGreen stain (Biotium, Hayward, CA). Positive amplicons were purified using a QIAquick PCR Purification Kit (Qiagen) and sequenced in both directions using an ABI 3130 Genetic Analyzer (Applied Biosystems, Foster City, CA). Forward and reverse reads were assembled and edited using the SeqMan Pro program in Lasergene v14 (DNASTAR, Madison, WI). Sequences obtained from bat blood or bat flies were initially confirmed to the bacterial genus using the Basic Local Alignment Search Tool (BLAST; <https://blast.ncbi.nlm.nih.gov/Blast.cgi>).

*Phylogenetic analysis of bacterial sequences*

Bacterial sequences obtained from bats and bat flies were aligned with reference sequences for named *Bartonella* species and with sequences that have been detected previously in bats or bat flies (Supplementary Data). Alignments for each genetic locus were performed separately using MAFFT v7.471 (Katoh and Standley, 2013) using the L-INS-i method. Alignments were trimmed to a common length and poorly aligned positions were eliminated using Gblocks v0.91b (Castresana, 2000)⁠. Alignments were then visually inspected for errors and manually corrected. Concatenation of multiple loci for phylogenetic analysis was performed after alignment and trimming using Phyutility v2.7.1 (Smith and Dunn, 2008). Simultaneous model selection and maximum likelihood reconstruction of phylogenetic trees was performed using IQ-TREE v2.1.1 (Kalyaanamoorthy *et al.*, 2017; Minh *et al.*, 2020). Branch support for each tree was estimated from 1000 ultrafast bootstrap samples from the respective alignment (Hoang *et al.*, 2018).

*Regression analyses*

Linear regression was performed to determine demographic factors that influence *Bartonella* infection status for bats sampled from the colony in March 2010. Two data sets were used: a set containing data from all bats and a set containing data from females only. In the full data set, covariates included bat sex, age class (neonate, juvenile, sexually immature adult, and sexually mature adult) following Peel *et al.* (2016), and the presence/absence of bat flies on each bat. In the females only data set, covariates were the same as the full dataset (excepting sex) but also included pregnancy status (pregnant or not). Data were fit to covariates in a generalized linear model (GLM), treating infection status as a binomial variable with a logit link. Model selection was then performed based on the Akaike information criterion corrected for finite sample sizes (AICc) using the dredge function in the R package *MuMIn* (Bartón, 2024; R Core Team, 2025). The model with the smallest AICc was chosen unless another model was less than two AICc points away from the top model (Burnham and Anderson, 2004), in which case the simpler model was chosen. Additional GLMs were fit to examine the effect of restocking flies on *Bartonella* infection in individual bat flies and prevalence, including whether bats received flies on 17 January 2012 or not, age, and sex as predictors.

Segmented regression was performed to detect breakpoints in measures of *Bartonella* prevalence and diversity over the course of the experiment. Separate GLMs were fit for each measure in R. *Bartonella* single infection, coinfection prevalence, and beta diversity (Jaccard index for presence/absence data) were treated as binomial variables with a logit link. Real-time PCR cycle threshold (Ct) values, Shannon number, and inverse Simpson index were treated as gamma-distributed variables. *Bartonella* species richness and the number of *Bartonella* species in a sample were treated as Poisson-distributed variables. Segmented regression was performed on fitted GLMs using the R package *segmented* (Muggeo, 2003, 2024) with breakpoints estimated with 1000 bootstrap iterations.

*Bayesian estimation of infection durations*

Appropriate statistical distributions for *Bartonella* infection durations were selected by model selection. Five one- or two-parameter, positive and continuous candidate distributions were tested: lognormal, inverse Gaussian, gamma, Weibull, and exponential. Models were fit using maximum likelihood and ranked according to the Akaike information criterion (AIC). The lognormal distribution was the top model according to AIC. The *stanarm* package was used to estimate separate means durations for each *Bartonella* species, fitting a Gaussian model to log duration (Carpenter *et al.*, 2017; Gabry *et al.*, 2025). Default priors for lognormal distributions were used. Mean estimates and 95% posterior intervals were generated from four chains of 2000 iterations, discarding the first 1000 iterations as burn-in. Convergence of chains was confirmed standard MCMC diagnostics: Monte Carlo standard error, the potential scale reduction factor (i.e., the Gelman-Rubin diagnostic, Rhat), and the effective sample size (Roy, 2020).

*Likelihood ratio tests*

We tested whether *Bartonella* species coinfected bats with other species more than expected by chance using a multinomial test adapted from a previous study analysing patterns of influenza A transmission in birds (Pepin *et al.*, 2013). Following Pepin et al., only double infections (two coinfecting species) were included because higher-order infections were rare and challenging to interpret. The null hypothesis for the test was that *Bartonella* species $i$ would coinfect with any other species $j$ with equal probability, and the expected counts for partner coinfections of species $i$ would be proportional to the frequency of each partner in all single (*s*) and double (*d*) infections. Therefore, the expected counts for each $j$ partner of species $i$, $E\left[ X_{j} \right]$ are: $E\left[ X_{j} \right] =\left( {X_{j}^{s+d}}/{N_{j}^{s+d}} \right) * X_{i}^{d}$, where $X_{j}^{s+d}$ is the total number of single and double infections for partner $j$, $N_{j}^{s+d}$ is the total number of single and double infections for all $j$ partners, and $X_{i}^{d}$ is the total number of double infections for species $i$. The maximum likelihood estimates for the parameters in the null multinomial model for each species $i$ are then: ${\pi_{j} = E\left[ X_{1} \right]}/{\sum\left( E\left[ X_{j} \right] \right)},\ldots, {E\left[ X_{N} \right]}/{\sum\left( E\left[ X_{J} \right] \right)}$. The probabilities under the null and alternative models are: $P\left( X \right)_{0} = N_{j}^{d}!\prod\left( {\pi_{j}^{xi}}/{X_{i}!} \right)$ and $P\left( X \right)_{A} = N_{j}^{d}!\prod\left( {p_{j}^{xi}}/{X_{i}!} \right)$ and the likelihood ratio statistic $D$ is $-\ln\left( {P\left( X \right)_{0}}/{P\left( X \right)_{A}} \right)$, which is approximately distributed $\chi_{n-1}^{2}$. The likelihood ratio statistic was divided by the correction factor $1+ \sum\left( \pi_{j}^{-1}-1 \right)/{6N_{j}^{d}\left( n-1 \right)}$ to decrease type I error inflation due to the difference between the moments of the likelihood ratio statistic and the chi-square distribution. Differences between the observed and expected counts of coinfections were tested using binomial likelihood ratio tests, using the same correction factor as above. Functions for multinomial and binomial likelihood ratio tests were written in R. These functions were first used to test for differences in observed and expected counts of coinfections for the whole course of the experiment (961 days). Additional tests were performed on two partitions of the experiment: bats sampled before 17 January 2012 and bats sampled after that date. This was based on visual observation of a change in the frequency of *Bartonella* species starting around this point in the experiment.

In addition to the tests of the observed and expected counts of coinfections, these same likelihood ratio test functions were used to perform tests on changes in the frequency of single infections and coinfections in the captive colony over time and differences in the relative frequency of infections between bats and bat flies. Specifically, we performed likelihood ratio tests on the relative frequency of *Bartonella* species before versus after 17 January 2012, using the frequencies before this date as the expected frequencies to calculate the likelihood ratio statistic. We calculated the differences in the frequency of *Bartonella* species in bats versus bat flies sampled in March 2010 and January 2012, using the frequencies in bats as the expected frequencies. We also calculated the differences in the frequencies of *Bartonella* species in bats after 17 January 2012 versus bat flies sampled on that date, again using the frequencies in bats as the expected frequencies.

*Assumptions*

Within this system, *Bartonella* infection does not cause obvious signs of disease in bats or flies (Kosoy *et al.*, 2010), so we assume that there are no parasite-mediated mortality effects. We consider hosts as discrete patches containing parasite species and the dynamics of these infections are linked through transmission by bat flies as they disperse among hosts. We consider *Bartonella* species as static and not measurably evolving over the current study, an assumption supported by the very low mutation rates (Gutiérrez *et al.*, 2018). Finally, although vertical transmission of *Bartonella* from dam to offspring is possible (Kosoy *et al.*, 1998) ⁠, it has not been demonstrated in bats, so we assume that bats are born uninfected, and the primary transmission route is through vector-borne transmission.

**Appendix 2. Supplementary results**

*Phylogenetic analysis of detected* Bartonella *species*

*Bartonella* sequences from *E. helvum* and *C. greefi* predominantly grouped closely with six *Bartonella* species previously described from *E. helvum*: *Bartonella* spp. E1–E5 and Ew (Kosoy *et al.*, 2010; Bai *et al.*, 2015). The phylogenetic distinctiveness of these species can be observed based on all loci sequenced: *gltA*, *ftsZ*, and ITS (Figures S1–S3). In addition to these six species, two novel *Bartonella* genogroups were observed in both *E. helvum* and *C. greefi*, denoted *Bartonella* spp. Eh6 and Eh7. *Bartonella* sp. Eh6 was detected at all three sequenced loci, whereas species Eh7 was only detected at *gltA* and *ftsZ* (Figures S1–S3). All sequences from bats and bat flies clustered with one of these eight *Bartonella* clades when separate maximum likelihood trees were generated for each locus (data not shown). Sequences representing *Bartonella* species Eh6 and Eh7 have been submitted to GenBank with the following accession numbers: MN250730–MN250774 (*gltA*), MN250775–MN250788 (*ftsZ*), and MN249715–MN249720 (ITS).

All *gltA* sequences from species Eh6 were found to be similar to each other (88.5–100% sequence identity), and according to BLAST search, similar (88.2–94.7% sequence identity) to a sequence obtained from *C. greefi* collected from *E. helvum* on Bioko island in the Gulf of Guinea (GenBank accession number JN172066) by (Billeter *et al.*, 2012). All *ftsZ* sequences were highly similar to each other (98.5–100% sequence identity), as were ITS sequences (99.4–100% sequence identity). All *gltA* sequences from species Eh7 were similar to one another (99.7–100% sequence identity) and similar (99.7–100% sequence identity) to six sequences (GenBank accession numbers JN172046, JN172050, JN172053, JN172058, JN172067, and JN172072) from *C. greefi* collected from *E. helvum* on Annobón and Bioko islands in the Gulf of Guinea and in Ghana (Billeter *et al.*, 2012)⁠. Sequenced loci grouped species Eh6 and Eh7 as monophyletic groups distinct from other *E. helvum*-associated *Bartonella* species with strong bootstrap support (Figures S1–S3).

A maximum likelihood tree produced from concatenated *ftsZ* and *gltA* sequences from known *Bartonella* species and *Bartonella* strains detected in bats (Supplementary Data) demonstrates that *Bartonella* species from *E. helvum* and *C. greefi* are broadly distributed in the *Bartonella* phylogeny (Figure S4). *Bartonella* sp. Ew clusters with 100% bootstrap support with three other *Bartonella* strains isolated from *Myotis blythii* and *Rhinolophus ferrumequinum* in Georgia (Urushadze *et al.*, 2017). *Bartonella* spp. E3, E1, E2, and E5 are part of a large and distinct clade of bat-associated *Bartonella* strains isolated from hosts in several bat families, including Hipposideridae, Rhinolophidae, Miniopteridae, Emballonuridae, and Vespertilionidae in Africa and Eurasia (Kosoy *et al.*, 2010; Lin *et al.*, 2012; Lilley *et al.*, 2015; McKee *et al.*, 2017; Urushadze *et al.*, 2017)⁠. While this clade only received 75% bootstrap support in the current tree using concatenated *ftsZ* and *gltA*, a previous analysis using three additional loci and a Bayesian phylogenetic approach found 100% posterior support for this clade (McKee *et al.*, 2017). *Bartonella* sp. Eh6 forms a clade including strains from *Pipistrellus pipistrellus* and *Myotis blythii* from Georgia (Urushadze *et al.*, 2017) with 97% bootstrap support. The Bayesian analysis by McKee *et al.* (2017) showed that this smaller clade is included as a subclade within the larger Old World bat-associated clade mentioned above with 100% posterior support. *Bartonella* spp. E4 and Eh7 were found to be most closely related to each other, although with only 51% bootstrap support. These two species are included in a larger clade containing *Bartonella* strains associated with rodents, carnivores, marsupials, and another bat (*Myotis emarginatus*) from Georgia (Urushadze *et al.*, 2017). While the bootstrap support for this larger clade is low (58%), sequencing of additional markers may result in higher support (McKee *et al.*, 2017; Urushadze *et al.*, 2017).

*Effects of bat fly restocking on treatment versus control bats*

There was poor correspondence between the *Bartonella* species found in the colony bats that received flies with the *Bartonella* species found in the bats that were the donors for the flies or other flies that were removed from the donor bats. The frequency of finding the same *Bartonella* species in the recipient bat and either the donor bat or a sampled fly taken from the donor bat (13/27, 48.1%) was no better than random (χ^2^ = 5E-31, df = 1, P = 0.5). This was again true if bats were subdivided into sexually immature and sexually mature adults (5/13, 38.5%; χ^2^ = 0.039, df = 1, P = 0.58) and neonates and juveniles (8/14, 57.1%; χ^2^ = 2.8E-32, df = 1, P = 0.5). Using the additional data from the collection of bat flies in March 2010, we also observed no correlation between the presence of a fly and whether a bat was positive (Pearson’s R = -0.067, t = -0.52, df = 59, P = 0.61). The frequency of finding the same *Bartonella* species in the bat and the sampled bat fly (9/26, 34.6%) was no better than random (χ^2^ = 0.71, df = 1, P = 0.8).

*Differences in* Bartonella *prevalence and diversity between bats and bat flies*

*Bartonella* prevalence in bat flies (93%) collected from the colony in March 2010 was similarly high as in the colony bats (Figure 3). The flies collected in January 2012 from the wild bat population had a slightly lower infection prevalence (89%) compared to the wild bats (94%), and both the wild flies and wild bats had higher prevalence than the bats in the colony (31%) on the same date. Average infection loads in flies in March 2010 were less than in the colony bats, indicated by higher Ct values (Figure S5). Similarly, wild bat flies had higher Ct values in January 2012 than the wild bats but were lower than in the colony bats. Bat flies had a lower coinfection prevalence compared to bats from their respective populations in the colony in March 2010 and from the wild population in January 2012 (Figure S9).

In March 2010, all *Bartonella* diversity measures (beta diversity, species richness, Shannon index, inverse Simpson index, and number of species in an individual sample) in flies were lower than in the bat population at that time (Figures S10 & S11). In January 2012, all diversity measures except the inverse Simpson index in wild bats were higher than in the captive colony. Diversity measures in flies sampled from wild bats at this time were lower than in the wild population.

There were significant differences in the frequencies of *Bartonella* species in the bats and flies sampled in March 2010 (D = 14.9, df = 7, P = 0.038), with significant differences observed in species E4 and E5 using binomial LR tests (Figure 4C; Table S9). Differences between the relative abundance of *Bartonella* species after the restocking of flies in January 2012 and the wild flies that were added to the colony were observed (D = 43.3, df = 6, P < 0.001), with substantially higher frequencies of E4 and Eh6 and lower abundance of E1 and E5 in the flies than the colony bats (Figure 4D; Table S9). Significant differences were not observed in the distribution of species between the wild bats and wild flies sampled in January 2012 (D = 5.8, df = 7, P = 0.57) (Figure 4D; Table S9).

*Individual infection histories and duration of infections*

Individual infection histories for all 112 identified bats and relevant statistics for their histories are included in the Supplementary Data. Out of the 112 individual bats sampled during this study period, 102 (91.1%) were sampled at least two time points in a row. The remaining 10 bats were either euthanized after the first sampling event (n = 1), were found dead between sampling time points (n = 5), or had disappeared and were presumed dead (n = 4).

There was considerable individual variation among bats in their infection histories, with some bats never becoming infected, bats with intermittent infections throughout the study, bats clearing infection soon after entry into the colony, and other bats with highly persistent infections. Of the 112 bats that were sampled, 100 (89.3%) tested positive at least once during the study and 65 (58%) bats were positive at entry into the colony. Of the 102 bats that were sampled more than once, 95 (93.1%) tested positive at least once: 80/95 (84.2%) were positive at more than one time point and 15/95 (15.8%) were positive only once. Of the 15 individuals positive only once, 12 (80%) were bats born into the colony in April 2010 (n = 2) or April 2011 (n = 10) and 10/12 (83.3%) became positive only after the flies were restocked in January 2012. The three adult bats only infected once were from the cohort that entered the colony in January 2010. One of these bats was positive on entry and was found dead in May 2010, another became positive shortly after entry in March 2010, and the third adult did not become positive until after the fly restocking.

**References**

**Bai, Y., Hayman, D. T. S., McKee, C. D. and Kosoy, M. Y.** (2015). Classification of *Bartonella* strains associated with straw-colored fruit bats (*Eidolon helvum*) across Africa using a multi-locus sequence typing platform. *PLOS Neglected Tropical Diseases* **9**, e0003478. doi: 10.1371/journal.pntd.0003478.

**Bai, Y., Gilbert, A., Fox, K., Osikowicz, L. and Kosoy, M.** (2016). *Bartonella rochalimae* and *B. vinsonii* subsp. *berkhoffii* in wild carnivores from Colorado, USA. *Journal of Wildlife Diseases* **52**, 844–849. doi: 10.7589/2016-01-015.

**Bartón, K.** (2024). MuMIn: multi-model inference.

**Billeter, S. A., Hayman, D. T. S., Peel, A. J., Baker, K., Wood, J. L. N., Cunningham, A., Suu-Ire, R., Dittmar, K. and Kosoy, M. Y.** (2012). *Bartonella* species in bat flies (Diptera: Nycteribiidae) from western Africa. *Parasitology* **139**, 324–329. doi: 10.1017/S0031182011002113.

**Birtles, R. J. and Raoult, D.** (1996). Comparison of partial citrate synthase gene (*gltA*) sequences for phylogenetic analysis of *Bartonella* species. *International Journal of Systematic and Evolutionary Microbiology* **46**, 891–897. doi: 10.1099/00207713-46-4-891.

**Burnham, K. P. and Anderson, D. R.** (2004). Multimodel inference: understanding AIC and BIC in model selection. *Sociological Methods & Research* **33**, 261–304. doi: 10.1177/0049124104268644.

**Carpenter, B., Gelman, A., Hoffman, M. D., Lee, D., Goodrich, B., Betancourt, M., Brubaker, M., Guo, J., Li, P. and Riddell, A.** (2017). Stan: a probabilistic programming language. *Journal of Statistical Software* **76**, 1–32. doi: 10.18637/jss.v076.i01.

**Castresana, J.** (2000). Selection of conserved blocks from multiple alignments for their use in phylogenetic analysis. *Molecular Biology and Evolution* **17**, 540–552. doi: 10.1093/oxfordjournals.molbev.a026334.

**Colborn, J. M., Kosoy, M. Y., Motin, V. L., Telepnev, M. V., Valbuena, G., Myint, K. S., Fofanov, Y., Putonti, C., Feng, C. and Peruski, L.** (2010). Improved detection of *Bartonella* DNA in mammalian hosts and arthropod vectors by real-time PCR using the NADH dehydrogenase gamma subunit (*nuoG*). *Journal of Clinical Microbiology* **48**, 4630–4633. doi: 10.1128/jcm.00470-10.

**Diaz, M. H., Bai, Y., Malania, L., Winchell, J. M. and Kosoy, M. Y.** (2012). Development of a novel genus-specific real-time PCR assay for detection and differentiation of *Bartonella* species and genotypes. *Journal of Clinical Microbiology* **50**, 1645–1649. doi: 10.1128/jcm.06621-11.

**Diniz, P. P. V. D. P., Maggi, R. G., Schwartz, D. S., Cadenas, M. B., Bradley, J. M., Hegarty, B. and Breitschwerdt, E. B.** (2007). Canine bartonellosis: serological and molecular prevalence in Brazil and evidence of co-infection with *Bartonella henselae* and *Bartonella vinsonii* subsp. *berkhoffii*. *Veterinary Research* **38**, 697–710. doi: 10.1051/vetres:2007023.

**Gabry, J., Ali, I., Brilleman, S., Novik, J. B., AstraZeneca, Trustees of Columbia University, Wood, S., R Core Development Team, Bates, D., Maechler, M., Bolker, B., Walker, S., Ripley, B., Venables, W., Burkner, P.-C. and Goodrich, B.** (2025). rstanarm: Bayesian applied regression modeling via Stan.

**Gundi, V. A. K. B., Kosoy, M. Y., Makundi, R. H. and Laudisoit, A.** (2012). Identification of diverse *Bartonella* genotypes among small mammals from Democratic Republic of Congo and Tanzania. *The American Journal of Tropical Medicine and Hygiene* **87**, 319–326. doi: 10.4269/ajtmh.2012.11-0555.

**Gutiérrez, R., Markus, B., Carstens Marques de Sousa, K., Marcos-Hadad, E., Mugasimangalam, R. C., Nachum-Biala, Y., Hawlena, H., Covo, S. and Harrus, S.** (2018). Prophage-driven genomic structural changes promote *Bartonella* vertical evolution. *Genome Biology and Evolution* **10**, 3089–3103. doi: 10.1093/gbe/evy236.

**Hoang, D. T., Chernomor, O., von Haeseler, A., Minh, B. Q. and Vinh, L. S.** (2018). UFBoot2: improving the ultrafast bootstrap approximation. *Molecular Biology and Evolution* **35**, 518–522. doi: 10.1093/molbev/msx281.

**Kalyaanamoorthy, S., Minh, B. Q., Wong, T. K. F., von Haeseler, A. and Jermiin, L. S.** (2017). ModelFinder: fast model selection for accurate phylogenetic estimates. *Nature Methods* **14**, 587–589. doi: 10.1038/nmeth.4285.

**Katoh, K. and Standley, D. M.** (2013). MAFFT multiple sequence alignment software version 7: improvements in performance and usability. *Molecular Biology and Evolution* **30**, 772–780. doi: 10.1093/molbev/mst010.

**Kosoy, M. Y., Regnery, R. L., Kosaya, O. I., Jones, D. C., Marston, E. L. and Childs, J. E.** (1998). Isolation of *Bartonella* spp. from embryos and neonates of naturally infected rodents. *Journal of Wildlife Diseases* **34**, 305–309. doi: 10.7589/0090-3558-34.2.305.

**Kosoy, M., Bai, Y., Lynch, T., Kuzmin, I. V., Niezgoda, M., Franka, R., Agwanda, B., Breiman, R. F. and Rupprecht, C. E.** (2010). *Bartonella* spp. in bats, Kenya. *Emerging Infectious Diseases* **16**, 1875–1881. doi: 10.3201/eid1612.100601.

**Lilley, T. M., Veikkolainen, V. and Pulliainen, A. T.** (2015). Molecular detection of *Candidatus* Bartonella hemsundetiensis in bats. *Vector-Borne and Zoonotic Diseases* **15**, 706–708. doi: 10.1089/vbz.2015.1783.

**Lin, J.-W., Hsu, Y.-M., Chomel, B. B., Lin, L.-K., Pei, J.-C., Wu, S.-H. and Chang, C.-C.** (2012). Identification of novel *Bartonella* spp. in bats and evidence of Asian gray shrew as a new potential reservoir of *Bartonella*. *Veterinary Microbiology* **156**, 119–126. doi: 10.1016/j.vetmic.2011.09.031.

**McKee, C. D., Kosoy, M. Y., Bai, Y., Osikowicz, L. M., Franka, R., Gilbert, A. T., Boonmar, S., Rupprecht, C. E. and Peruski, L. F.** (2017). Diversity and phylogenetic relationships among *Bartonella* strains from Thai bats. *PLOS ONE* **12**, e0181696. doi: 10.1371/journal.pone.0181696.

**Minh, B. Q., Schmidt, H. A., Chernomor, O., Schrempf, D., Woodhams, M. D., von Haeseler, A. and Lanfear, R.** (2020). IQ-TREE 2: new models and efficient methods for phylogenetic inference in the genomic era. *Molecular Biology and Evolution* **37**, 1530–1534. doi: 10.1093/molbev/msaa015.

**Muggeo, V. M. R.** (2003). Estimating regression models with unknown break-points. *Statistics in Medicine* **22**, 3055–3071. doi: 10.1002/sim.1545.

**Muggeo, V. M. R.** (2024). segmented: regression models with break-points/change-points estimation (with possibly random effects).

**Norman, A. F., Regnery, R., Jameson, P., Greene, C. and Krause, D. C.** (1995). Differentiation of *Bartonella*-like isolates at the species level by PCR-restriction fragment length polymorphism in the citrate synthase gene. *Journal of Clinical Microbiology* **33**, 1797–1803. doi: 10.1128/jcm.33.7.1797-1803.1995.

**Peel, A. J., Baker, K. S., Hayman, D. T. S., Suu-Ire, R., Breed, A. C., Gembu, G.-C., Lembo, T., Fernández-Loras, A., Sargan, D. R., Fooks, A. R., Cunningham, A. A. and Wood, J. L. N.** (2016). Bat trait, genetic and pathogen data from large-scale investigations of African fruit bats, *Eidolon helvum*. *Scientific Data* **3**, 160049. doi: 10.1038/sdata.2016.49.

**Pepin, K. M., Wang, J., Webb, C. T., Smith, G. J. D., Poss, M., Hudson, P. J., Hong, W., Zhu, H., Riley, S. and Guan, Y.** (2013). Multiannual patterns of influenza A transmission in Chinese live bird market systems. *Influenza and Other Respiratory Viruses* **7**, 97–107. doi: 10.1111/j.1750-2659.2012.00354.x.

**R Core Team** (2025). R: a language and environment for statistical computing.

**Roy, V.** (2020). Convergence diagnostics for Markov chain Monte Carlo. *Annual Review of Statistics and Its Application* **7**, 387–412. doi: 10.1146/annurev-statistics-031219-041300.

**Smith, S. A. and Dunn, C. W.** (2008). Phyutility: a phyloinformatics tool for trees, alignments and molecular data. *Bioinformatics* **24**, 715–716. doi: 10.1093/bioinformatics/btm619.

**Urushadze, L., Bai, Y., Osikowicz, L., McKee, C., Sidamonidze, K., Putkaradze, D., Imnadze, P., Kandaurov, A., Kuzmin, I. and Kosoy, M.** (2017). Prevalence, diversity, and host associations of *Bartonella* strains in bats from Georgia (Caucasus). *PLOS Neglected Tropical Diseases* **11**, e0005428. doi: 10.1371/journal.pntd.0005428.

**Zeaiter, Z., Liang, Z. and Raoult, D.** (2002). Genetic classification and differentiation of *Bartonella* species based on comparison of partial *ftsZ* gene sequences. *Journal of Clinical Microbiology* **40**, 3641–3647. doi: 10.1128/jcm.40.10.3641-3647.2002.

**
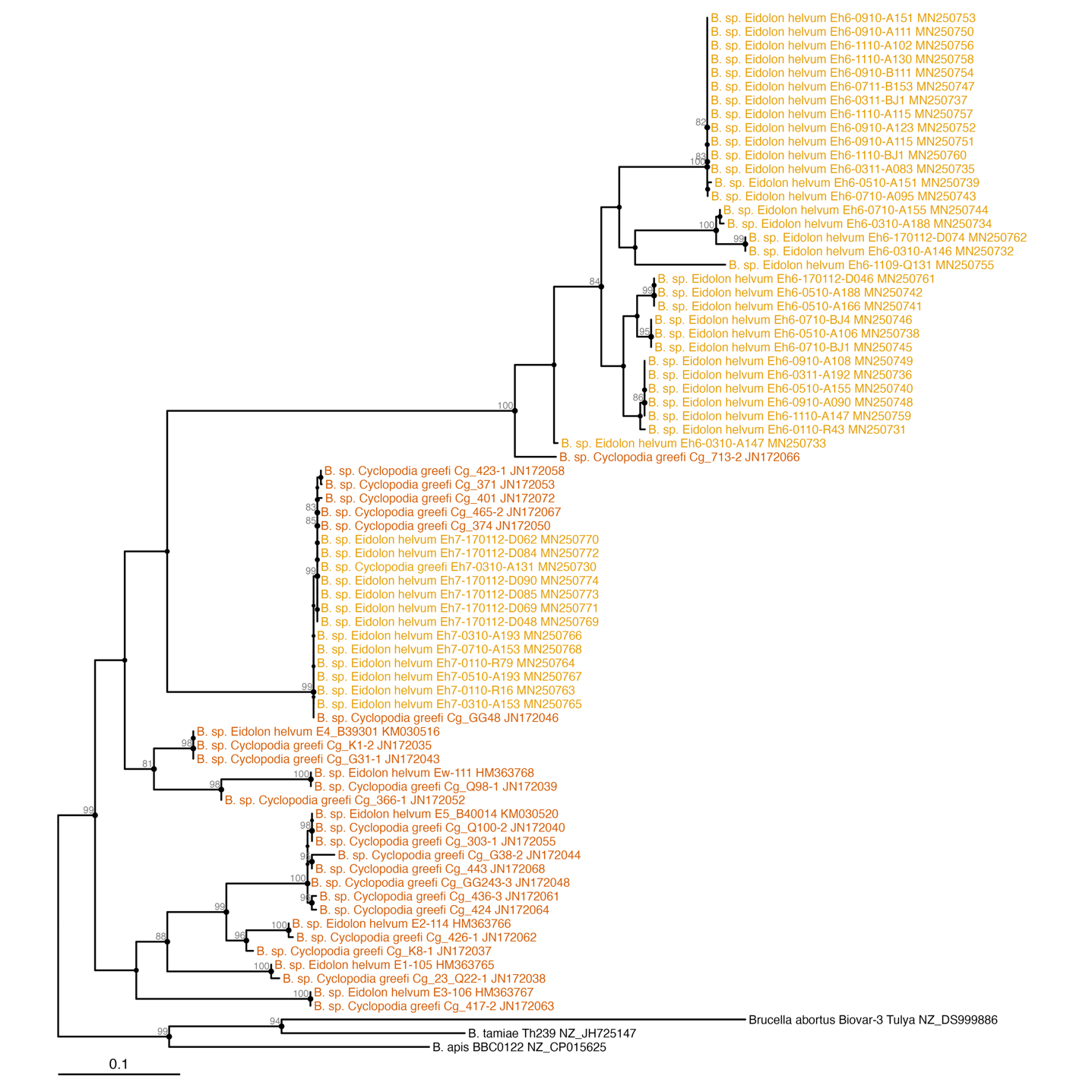
**

**Figure S1.** Maximum likelihood phylogenetic tree of *Bartonella* *gltA* sequences produced from a 356 bp alignment of 76 sequences. The best model of sequence evolution was a transition model with unequal base frequencies, a proportion of invariable sites, and four Gamma rate categories (TIM3+F+I+G4) based on AICc. The tree was rooted at the midpoint and bootstrap branch support values greater than 80% are shown in gray next to branches. Names of *Bartonella* sequences previously obtained from *E. helvum* or *C. greefi* are coloured orange, while names of new sequences from these species are coloured yellow.


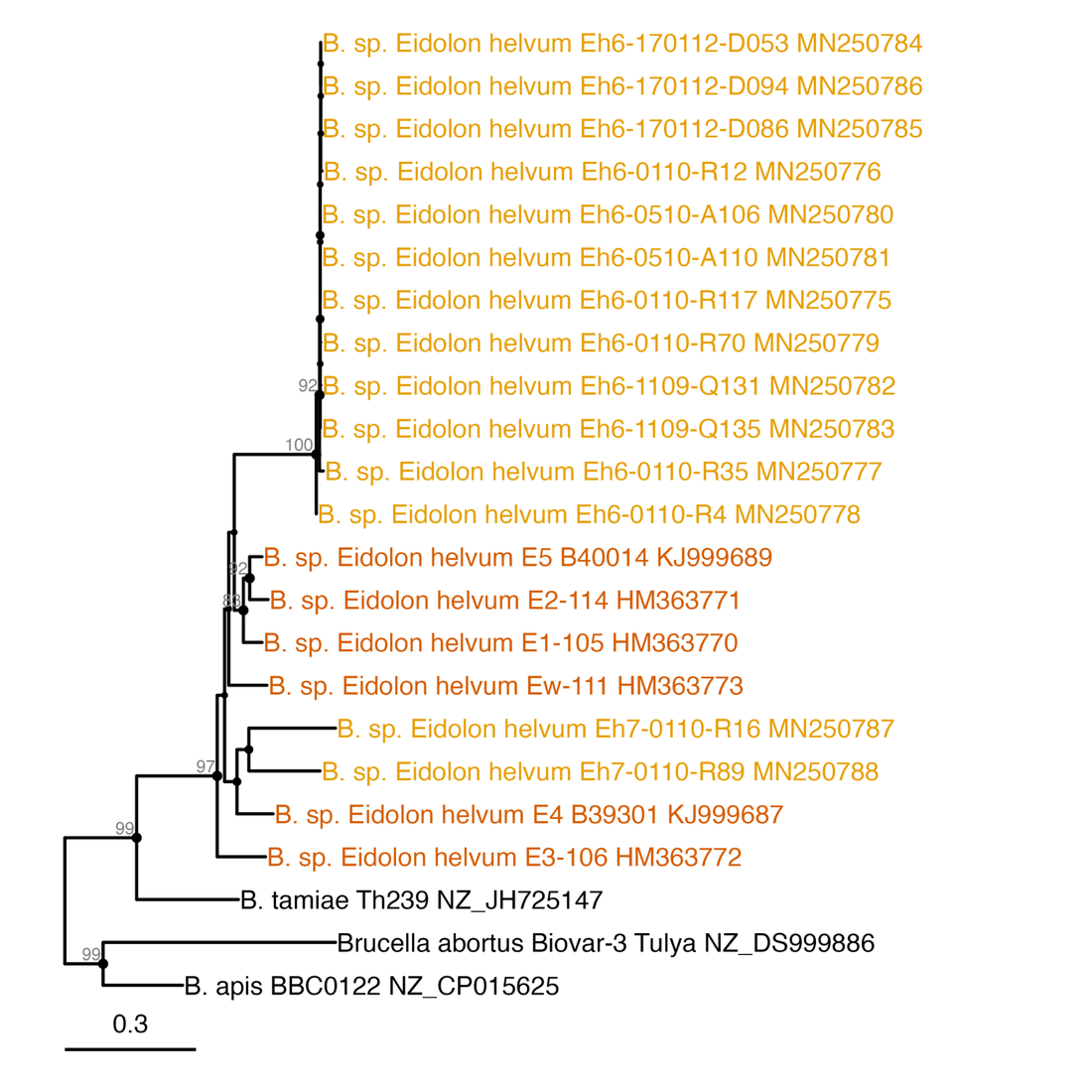


**Figure S2.** Maximum likelihood phylogenetic tree of *Bartonella* *ftsZ* sequences produced from a 727 bp alignment of 23 sequences. The best model of sequence evolution was a transversion model with unequal base frequencies, a proportion of invariable sites, and four Gamma rate categories (TVM+F+I+G4) based on AICc. The tree was rooted at the midpoint and bootstrap branch support values greater than 80% are shown in gray next to branches. Names of *Bartonella* sequences previously obtained from *E. helvum* or *C. greefi* are coloured orange, while names of new sequences from these species are coloured yellow.


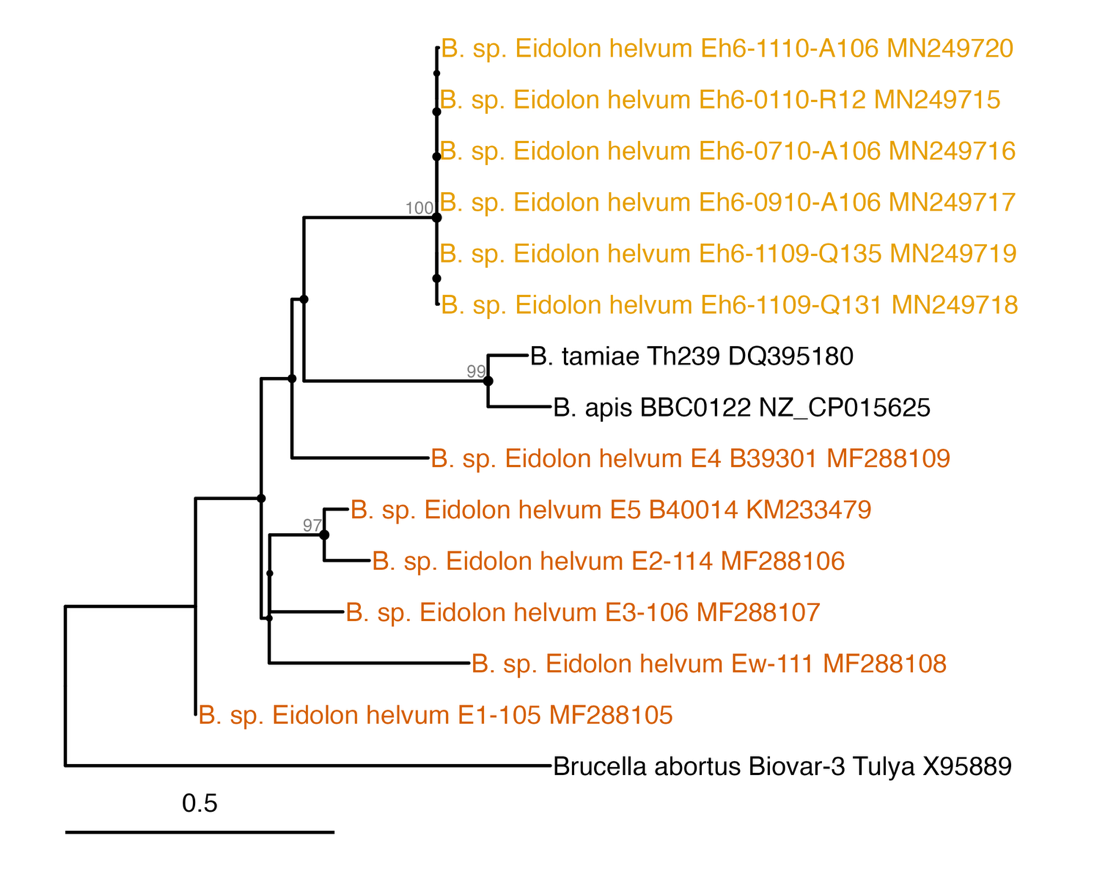


**Figure S3.** Maximum likelihood phylogenetic tree of *Bartonella* ITS sequences produced from a 446 bp alignment (including gaps) of 15 sequences. The best model of sequence evolution was a transition model with unequal base frequencies, a proportion of invariable sites, and four Gamma rate categories (TIM3+F+I+G4) based on AICc. The tree was rooted at the midpoint and bootstrap branch support values greater than 80% are shown in gray next to branches. Names of *Bartonella* sequences previously obtained from *E. helvum* or *C. greefi* are coloured orange, while names of new sequences from these species are coloured yellow.


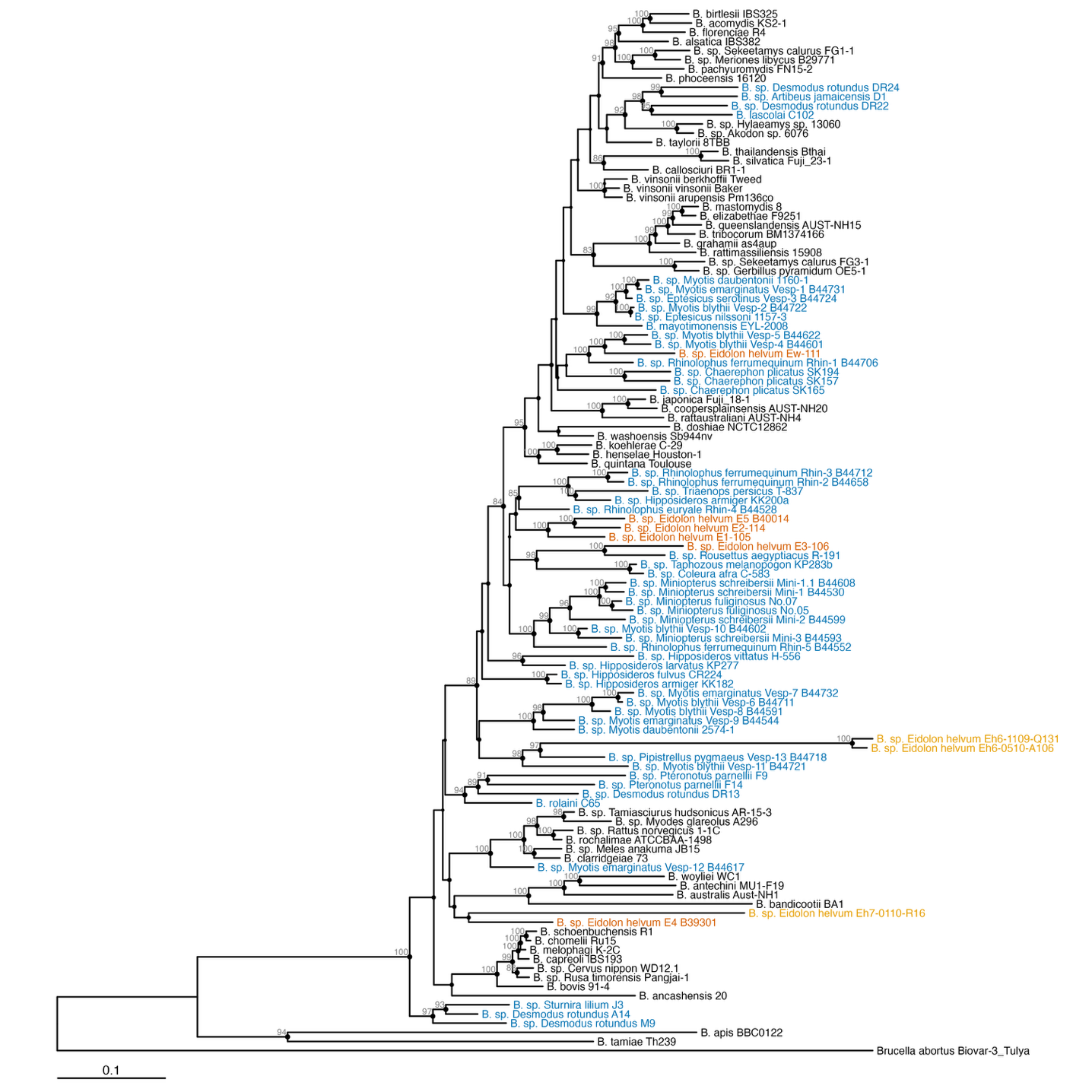


**Figure S4.** Maximum likelihood phylogenetic tree of concatenated *Bartonella* *ftsZ* and *gltA* sequences produced from a 1247 bp alignment (891 bp *ftsZ*, 356 bp *gltA*) of 114 sequences. The best model of sequence evolution was a transversion model with unequal base frequencies and seven free rate categories (TVM+F+R7) based on AICc. The tree was rooted at the midpoint and bootstrap branch support values greater than 80% are shown in gray next to branches. Names of *Bartonella* species/strains previously obtained from bats are coloured blue, sequences previously obtained from *E. helvum* or *C. greefi* are coloured orange, and names of new sequences from these species are coloured yellow.


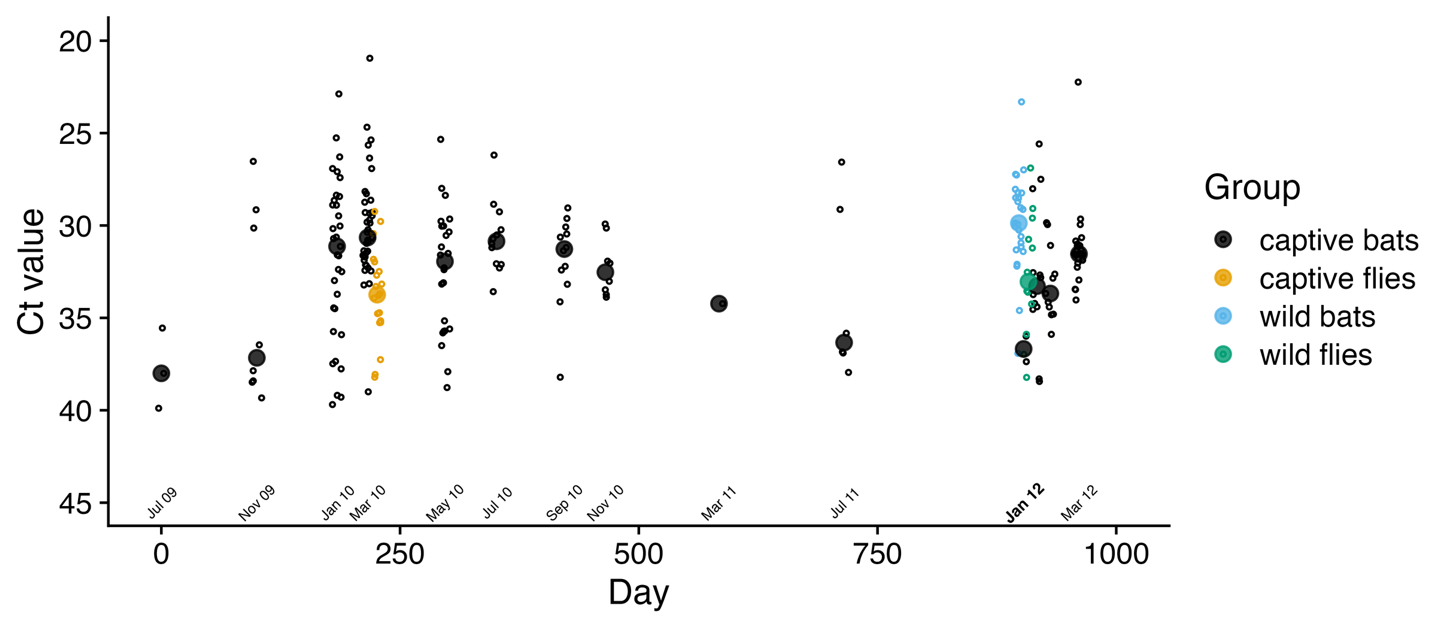


**Fig S5.** Bartonella infection load in a captive colony of *E. helvum* over time. Only points with real-time PCR Ct values < 40 are shown. Mean Ct values calculated at each time point are drawn as filled circles over the data (open circles). The month labelled in bold font on the x-axis shows when bat flies were added to the bat colony.


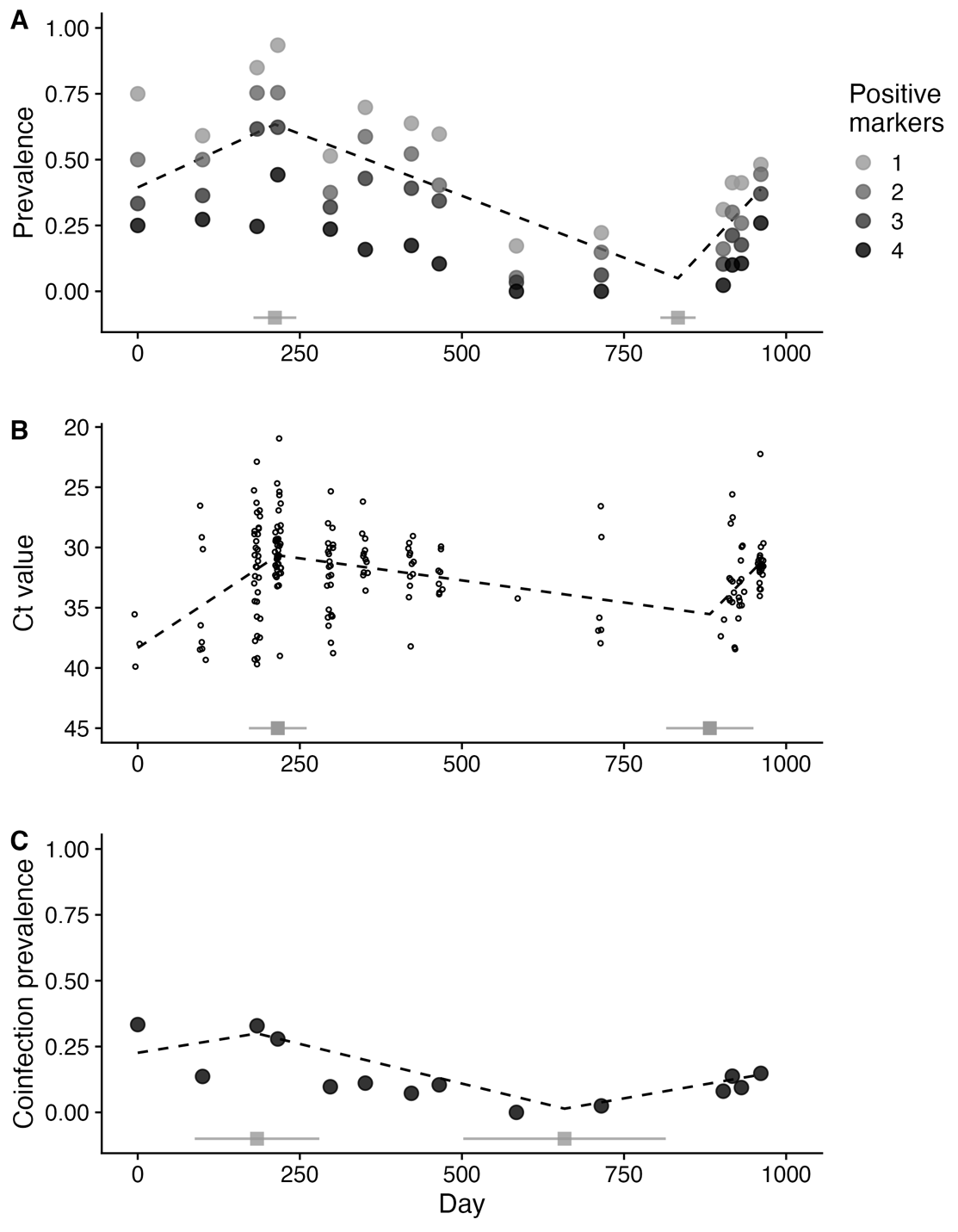


**Figure S6.** Segmented regression analysis of *Bartonella* prevalence and load. (A) Points for *Bartonella* prevalence are shown considering one or more, two or more, three or more, or all four markers positive (including real-time PCR). (B) Only points with real-time PCR Ct values < 40 are shown. (C) Coinfection prevalence was measured by the number of individuals that were positive for two or more *Bartonella* species at each time point. For each measure, dashed lines for the predicted trend from segmented regression are drawn over the data points. Breakpoints and 95% confidence intervals estimated by segmented regression are shown above the x-axis as grey squares and solid lines.

**
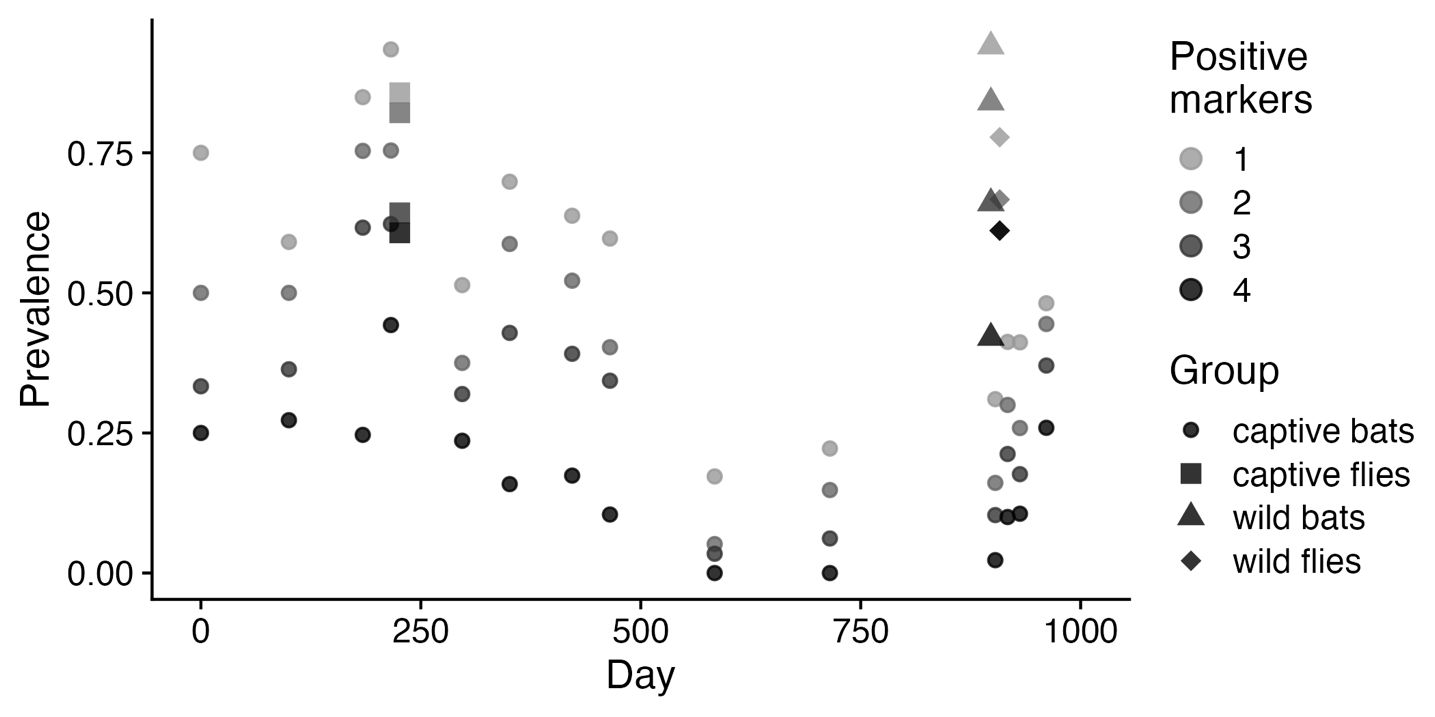
**

**Figure S7.** *Bartonella* infection prevalence according to the number of markers testing positive. Separate points are drawn for prevalence estimates in the *E. helvum* colony over time considering one or more, two or more, three or more, or all four markers positive (including real-time PCR). Points for sampled bat flies and wild bats are shown as unique symbols.


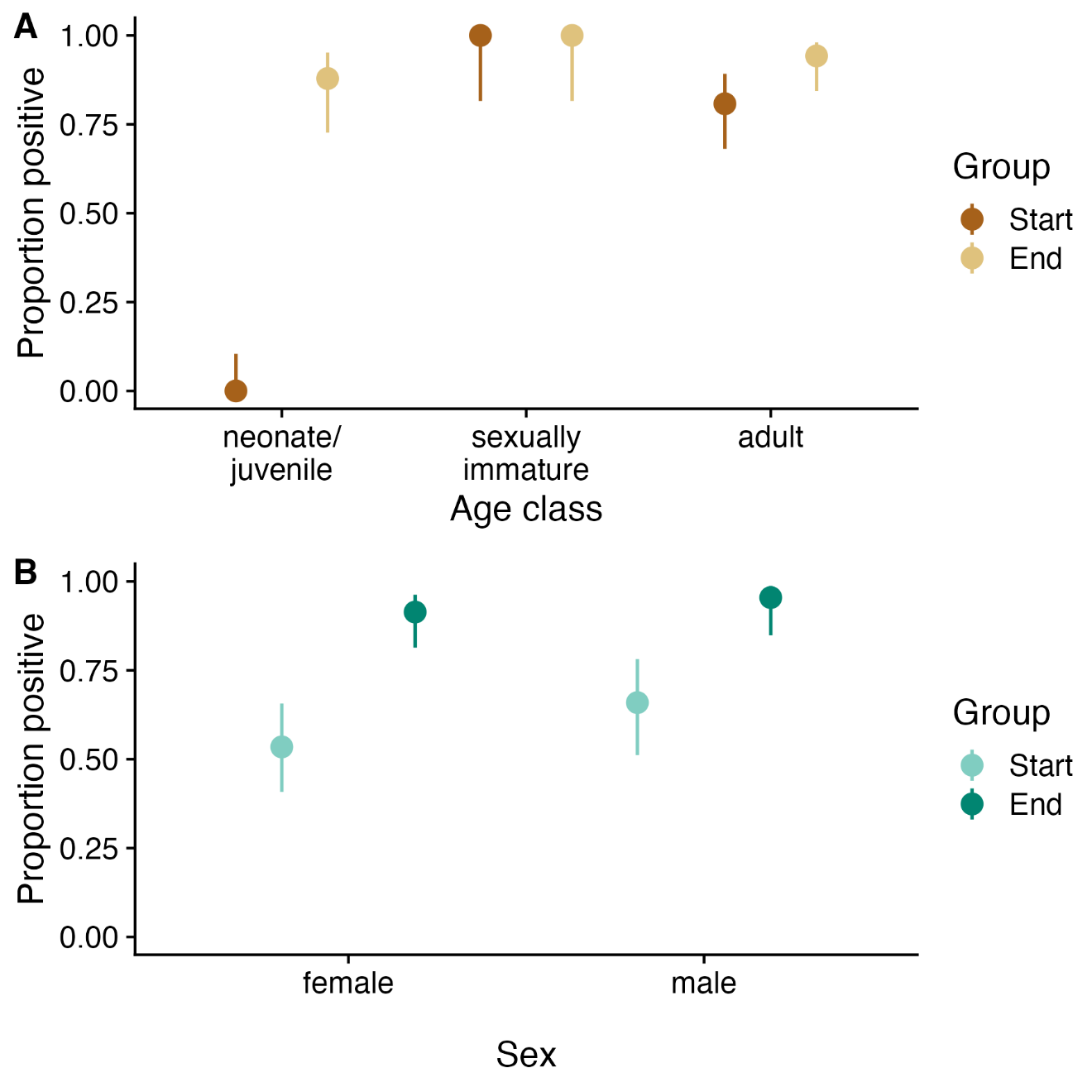


**Figure S8.** Change in the proportion of individuals positive for *Bartonella* at the start (upon entry into colony) and end (15 March 2012) of the experiment according to (A) age class and (B) sex. Wilson score 95% confidence intervals are included as lines.


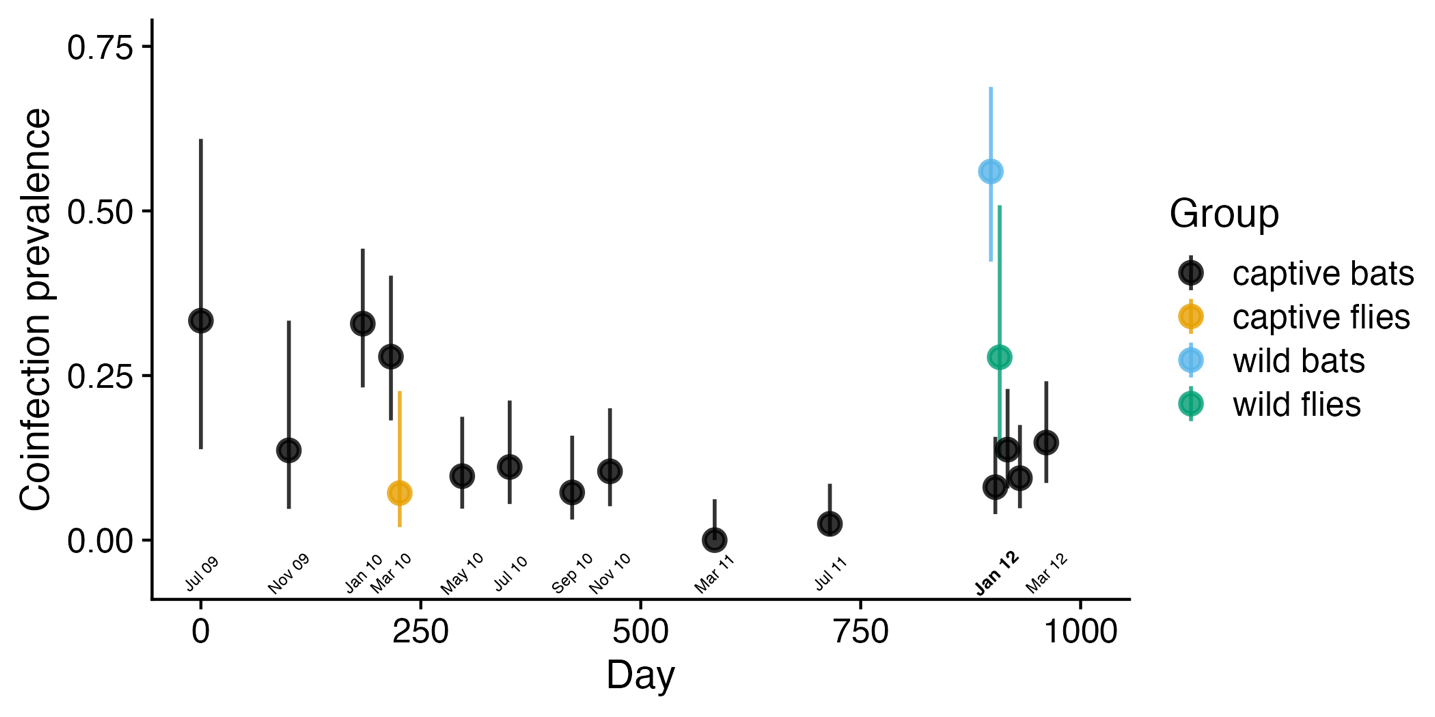


**Figure S9.** *Bartonella* coinfection prevalence in the *E. helvum* colony. Coinfection prevalence was measured by the number of bats and bat flies that were positive for two or more *Bartonella* species at each time point. Wilson score 95% confidence intervals were drawn around prevalence estimates at each sampling time point.

**
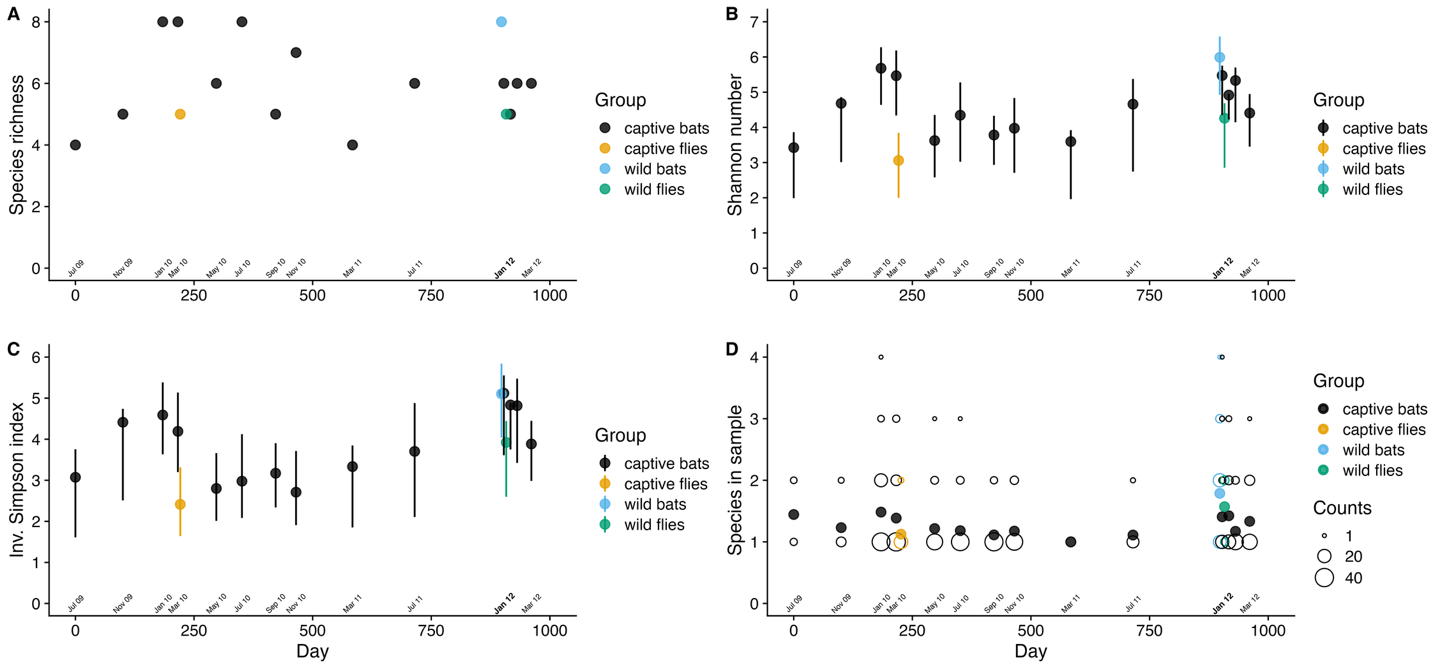
**

**Figure S10.** *Bartonella* infection diversity measures over time. Species richness (A), Shannon index of species evenness (B), and inverse Simpson index (C) are colony-level measures of *Bartonella* alpha diversity. The number of *Bartonella* species in each bat (D) is a measure of individual-level diversity. (B-C) Intervals around evenness indices are bootstrap 95% confidence intervals from 1000 samples from the observed multinomial distribution of *Bartonella* species relative abundances. (D) Points (open circles) show the number of *Bartonella* species observed for each individual with the width proportional to the number of individuals with that same number of *Bartonella* species. Mean values are drawn as filled circles. The month labelled in bold font on the x-axis shows when bat flies were added to the bat colony.


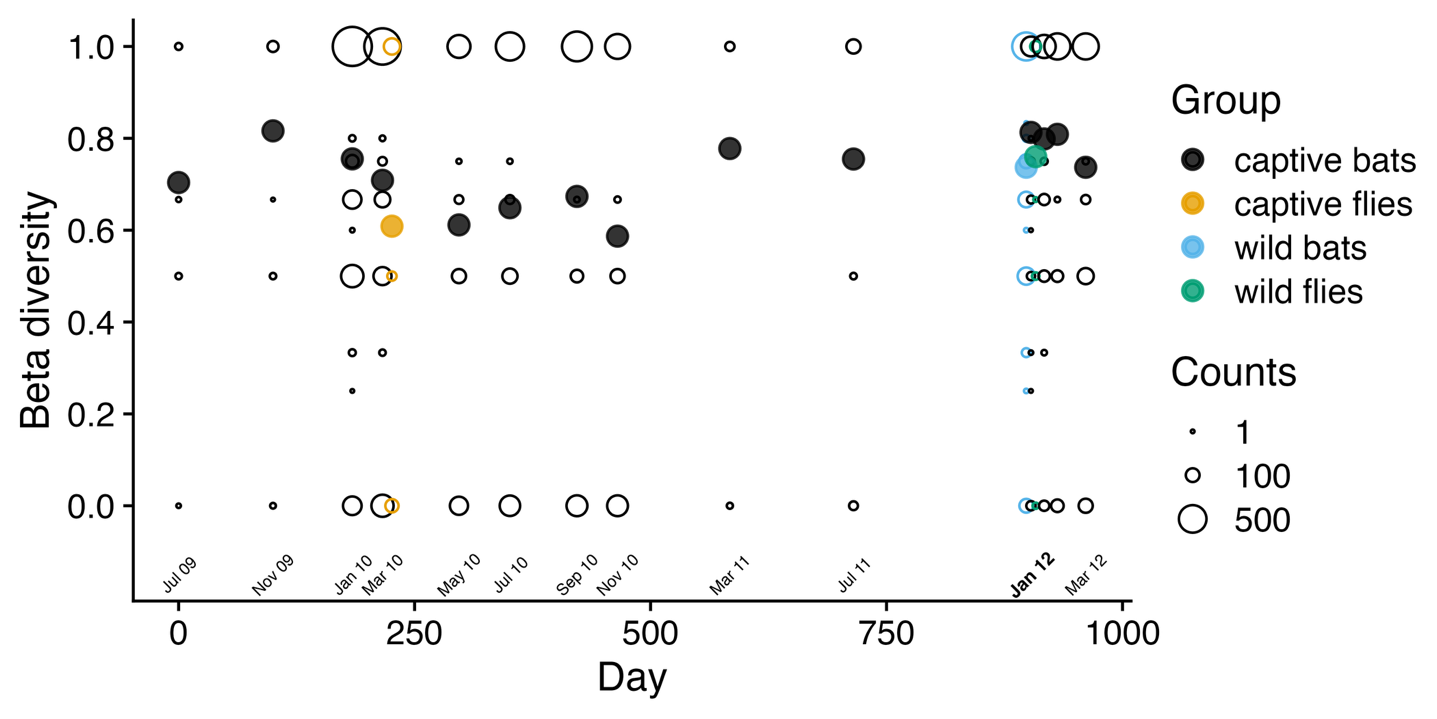


**Figure S11.** Changes in *Bartonella* beta diversity of *Bartonella* species in bats and bat flies over time. Beta diversity was calculated using the Jaccard index based on the presence/absence of *Bartonella* species, comparing across all infected bats and bat flies in the colony. Data for individuals are shown as open circles for each individual, with the width proportional to the number of individuals with the same index value. Solid circles show the mean values.


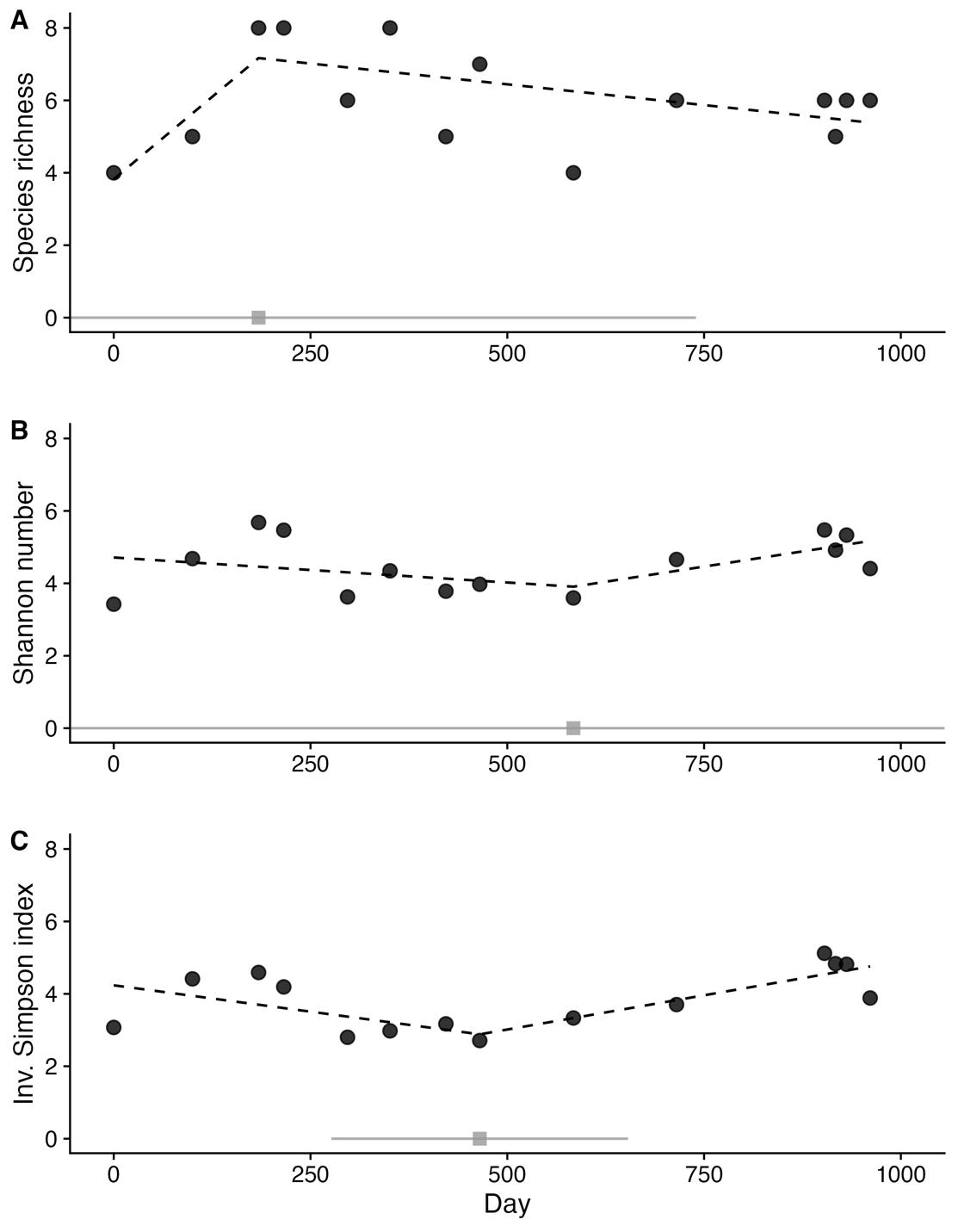


**Figure S12.** Segmented regression analysis of colony-level *Bartonella* diversity measures: (A) species richness, (B) Shannon index of species evenness, and (C) inverse Simpson index of species evenness. For each measure, dashed lines for the predicted trend from segmented regression are drawn over the data points. Breakpoints and 95% confidence intervals estimated by segmented regression are shown above the x-axis as grey squares and solid lines.


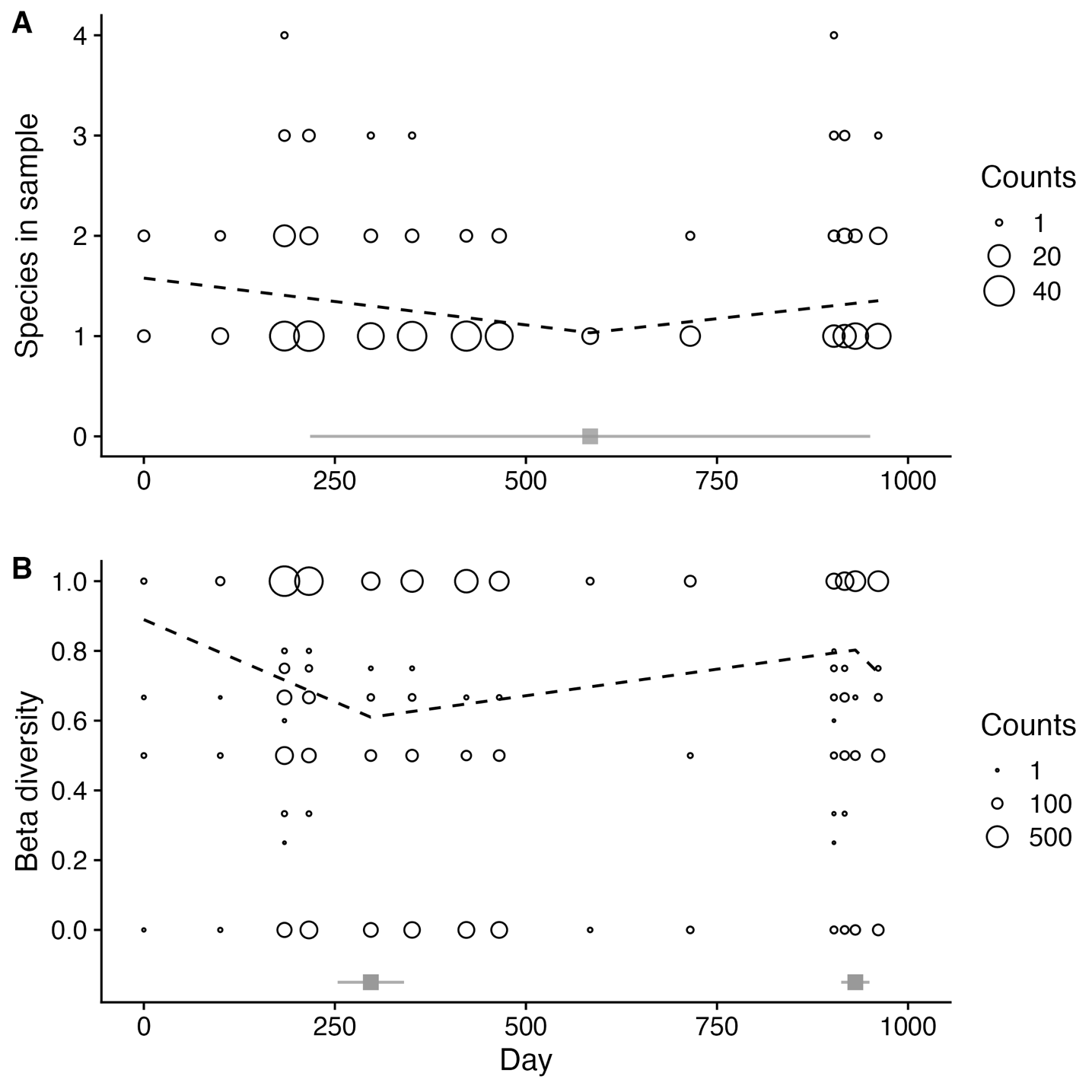


**Figure S13.** Segmented regression analysis of individual-level *Bartonella* diversity measures. Points show the number of *Bartonella* species observed in an individual sample (A) and the binomial index of beta diversity (B; compared to all other bats in the colony) for each individual with the width proportional to the number of individuals with that same diversity value. For each measure, dashed lines for the predicted trend from segmented regression are drawn over the data points. Breakpoints and 95% confidence intervals estimated by segmented regression are shown above the x-axis as grey squares and solid lines.

**Table S1.** Demographics of bats entering the captive colony (n = 112). Age classes follow Peel *et al.* (2016): neonate (NEO), juvenile (JUV), sexually immature adult (SI), and sexually mature adult (A). NEO and JUV classes were born in captivity (BIC) in 2010 and 2011 as cohorts 4 and 5.

|  | **Time (days) since** | | **Cohort number** | **A** | | **SI** | | **BIC (NEO/JUV)** | |
| --- | --- | --- | --- | --- | --- | --- | --- | --- | --- |
| **Date** | **Study start** | **Last sampling** |  | **F** | **M** | **F** | **M** | **F** | **M** |
| 2009-07-28 | 0 | 0 | 1 |  | 11 |  | 1 |  |  |
| 2009-11-05 | 100 | 100 | 2 | 3 | 5 | 3 | 2 |  |  |
| 2010-01-28 | 184 | 84 | 3 | 29 | 12 | 7 | 5 |  |  |
| 2010-03-06 | 221 | 37 |  |  |  |  |  |  |  |
| 2010-04-01 | 247 | No sampling | 4 |  |  |  |  | 7 | 4 |
| 2010-05-21 | 297 | 76 |  |  |  |  |  |  |  |
| 2010-07-14 | 351 | 54 |  |  |  |  |  |  |  |
| 2010-09-23 | 422 | 71 |  |  |  |  |  |  |  |
| 2010-11-05 | 465 | 43 |  |  |  |  |  |  |  |
| 2011-03-04 | 584 | 119 |  |  |  |  |  |  |  |
| 2011-04-01 | 612 | No sampling | 5 |  |  |  |  | 14 | 9 |
| 2011-07-13 | 715 | 131 |  |  |  |  |  |  |  |
| 2012-01-17 | 903 | 188 |  |  |  |  |  |  |  |
| 2012-01-31 | 917 | 14 |  |  |  |  |  |  |  |
| 2012-02-14 | 931 | 14 |  |  |  |  |  |  |  |
| 2012-03-15 | 961 | 30 |  |  |  |  |  |  |  |
|  |  |  |  | 32 | 28 | 10 | 8 | 21 | 13 |

**Table S2.** Oligonucleotide primers used for bacterial detection via real-time and conventional PCR amplification. Sequences designated [F] are forward primers, those designated [R] are reverse primers, and those designated [P] are TaqMan probes. FAM is 6-Carboxyfluorescein (maximum fluorescence at 518 nm).

| **Bacteria** | **Locus** | **PCR type** | **PCR round** | **Primer sequence** | **Primer name** | **Reference** |
| --- | --- | --- | --- | --- | --- | --- |
| *Bartonella* | *ftsZ* | conventional | 1 | ATTAATCTGCAY-  CGGCCAGA [F] | Bfp1 | (Zeaiter *et al.*, 2002) |
|  |  |  | 1 | ACVGADACACGA-  ATAACACC [R] | Bfp2 |  |
|  |  | conventional | 2 | ATATCGCGGAAT-  TGAAGCC [F] | ftsZ R83 | (Colborn *et al.*, 2010) |
|  |  |  | 2 | CGCATAGAAGTA-  TCATCCA [R] | ftsZ L83 |  |
| *Bartonella* | *gltA* | conventional | 1 | GCTATGTCTGCA-  TTCTATCA [F] | CS443f | (Birtles and Raoult, 1996; Gundi *et al.*, 2012) |
|  |  |  | 1 | GATCYTCAATCAT-  TTCTTTCCA [R] | CS1210r |  |
|  |  | conventional | 2 | GGGGACCAGCTC-  ATGGTGG [F] | BhCS781.p | (Norman *et al.*, 1995; Birtles and Raoult, 1996) |
|  |  |  | 2 | AATGCAAAAAGA-  ACAGTAAACA [R] | BhCS1137.n |  |
| *Bartonella* | ITS | conventional | 1 | CTTCAGATGATG-  ATCCCAAGCCTT-  CTGGCG [F] | 325s | (Diniz *et al.*, 2007) |
|  |  |  | 1 | GAACCGACGACC-  CCCTGCTTGCAA-  AGA [R] | 1100as |  |
| *Bartonella* | *ssrA* | real-time | 1 | GCTATGGTAATA-  AATGGACAATGA-  AATAA [F] | ssrA-F | (Diaz *et al.*, 2012) |
|  |  |  | 1 | GCTTCTGTTGCC-  AGGTG [R] | ssrA-R |  |
|  |  |  | 1 | (FAM)-  ACCCCGCTT-  AAACCTGCGACG-  (BHQ1) [P] | ssrA-P |  |

**Table S3.** Thermocycler protocols used for bacterial detection via real-time and conventional PCR amplification.

| **Bacteria** | **Locus** | **PCR type** | **PCR round** | **Thermal program** |
| --- | --- | --- | --- | --- |
| *Bartonella* | *ftsZ* | conventional | 1 | 95°C 4:00, (95°C 0:30, 55°C 0:30, 72°C 1:00)x40, 72°C 10:00, 4°C ∞ |
|  |  |  | 2 | 95°C 4:00, (95°C 0:30, 55°C 0:30, 72°C 1:00)x40, 72°C 10:00, 4°C ∞ |
| *Bartonella* | *gltA* | conventional | 1 | 95°C 2:00, (95°C 0:30, 48°C 0:30, 72°C 2:00)x40, 72°C 7:00, 4°C ∞ |
|  |  |  | 2 | 95°C 3:00, (95°C 0:30, 55°C 0:30, 72°C 0:30)x40, 72°C 7:00, 4°C ∞ |
| *Bartonella* | ITS | conventional | 1 | 95°C 3:00, (95°C 0:30, 66°C 0:30, 72°C 0:30)x55, 72°C 5:00, 4°C ∞ |
| *Bartonella* | *ssrA* | real-time | 1 | 60°C 1:00, 95°C 10:00, (95°C 0:15, 60°C 1:00)x45, 60°C 1:00, 4°C ∞ |

**Table S4.** Logistic regression results testing the effect of age class and sex on change in *Bartonella* infection status during the captive colony experiment (N = 112). In this model, the outcome of interest was whether a bat that had infection status measured multiple times during the study became *Bartonella*-positive by the end of the study after being initially negative at entry. Age classes follow Peel *et al.* (2016): neonate (NEO), juvenile (JUV), sexually immature adult (SI), and sexually mature adult (A). The intercept for the model used age class = NEO/JUV and sex = female as the reference categories. Model coefficients are shown in (A) while contrasts across age classes averaging across sex are shown in (B). P-values for contrasts shown in (B) have been adjusted for multiple comparisons using Tukey’s honestly significant difference test.

| **Outcome = whether a bat became *Bartonella*-positive after entry into the colony** | | | |
| --- | --- | --- | --- |
| **A** |  |  |  |
| **Term** | **Estimate (95% CI)** | **z** | **P** |
| (Intercept) | 2.11 (1.06, 3.47) | 3.51 | 0.00045 |
| Age = SI | -20.54 (-455.91, 57.76) | -0.01 | 0.99 |
| Age = A | -3.91 (-5.37, -2.7) | -5.84 | 5.3x10^-9^ |
| Sex = male | -0.34 (-1.68, 0.95) | -0.52 | 0.6 |
|  |  |  |  |
| **B** |  |  |  |
| **Contrast** | **Estimate** | **z ratio** | **P (Tukey-adjusted)** |
| (NEO/JUV) – SI | 20.54 | 0.01 | 1.0 |
| (NEO/JUV) – A | 3.91 | 5.84 | <0.0001 |
| SI – A | -16.63 | -0.01 | 1.0 |

**Table S5.** Logistic regression results testing the change in *Bartonella* infection status of bats present in the colony on 17 January 2012 and subsequent time points (N = 84). In one model, the outcome of interest was whether bats were *Bartonella*-positive at any time point after flies were restocked on 17 January 2012 (sections A and B). In the second model, the outcome was whether a bat became *Bartonella*-positive after 17 January 2012 (after being initially negative) or changed *Bartonella* species (if initially positive prior to 17 January 2012). Age classes follow Peel *et al.* (2016): neonate (NEO), juvenile (JUV), sexually immature adult (SI), and sexually mature adult (A). The intercept for both models used age class = NEO/JUV, sex = female, prior infection = 0, and received flies = 0 as the reference categories. Model coefficients are shown in sections A and C while contrasts across age classes averaging across other covariates are shown in sections B and D. P-values for contrasts shown in (B) have been adjusted for multiple comparisons using Tukey’s honestly significant difference test.

| **Outcome = whether a bat was *Bartonella­­*-positive at any point after 17 January 2012** | | | |
| --- | --- | --- | --- |
| **A** |  |  |  |
| **Term** | **Estimate (95% CI)** | **z** | **P** |
| (Intercept) | 0.94 (-0.12, 2.14) | 1.65 | 0.099 |
| Bat was infected prior to 17 January 2012 | 0.71 (-0.29, 1.77) | 1.36 | 0.17 |
| Age = SI | -1.76 (-3.55, -0.09) | -2.03 | 0.042 |
| Age = A | -1.69 (-3.05, -0.5) | -2.62 | 0.0088 |
| Sex = male | 0.47 (-0.57, 1.58) | 0.87 | 0.39 |
| Bat received flies on 17 January 2012 | 0.91 (-0.17, 2.06) | 1.61 | 0.11 |
|  |  |  |  |
| **B** |  |  |  |
| **Contrast** | **Estimate** | **z ratio** | **P (Tukey-adjusted)** |
| (NEO/JUV) – SI | 1.76 | 2.03 | 0.11 |
| (NEO/JUV) – A | 1.69 | 2.62 | 0.024 |
| SI – A | -0.07 | -0.11 | 0.99 |
|  |  |  |  |
| **Outcome = whether a bat became *Bartonella*-positive after 17 January 2012 (after being initially negative) or changed *Bartonella* species (if initially positive prior to 17 January 2012)** | | | |
| **C** |  |  |  |
| **Term** | **Estimate (95% CI)** | **z** | **P** |
| (Intercept) | 1.24 (0.19, 2.42) | 2.2 | 0.028 |
| Bat was infected prior to 17 January 2012 | -0.52 (-1.56, 0.49) | -1.0 | 0.32 |
| Age = SI | -1.06 (-2.72, 0.53) | -1.3 | 0.19 |
| Age = A | -2.09 (-3.39, -0.94) | -3.39 | 0.00071 |
| Sex = male | -0.11 (-1.13, 0.93) | -0.21 | 0.83 |
| Bat received flies on 17 January 2012 | 1.1 (0.05, 2.25) | 2.0 | 0.045 |
|  |  |  |  |
| **D** |  |  |  |
| **Contrast** | **Estimate** | **z ratio** | **P (Tukey-adjusted)** |
| (NEO/JUV) – SI | 1.06 | 1.3 | 0.39 |
| (NEO/JUV) – A | 2.09 | 3.39 | 0.002 |
| SI – A | 1.02 | 1.47 | 0.3 |

**Table S6.** Duration of *Bartonella* species infections in serially infected bats. For each *Bartonella* species, the counts record the individual bats that had the *Bartonella* species as their longest-lasting infection (i.e., the *Bartonella* species was present for the most sequential time points). Summary statistics are provided for the infection duration in days. Means and 95% posterior intervals were estimated using Bayesian lognormal regression. Convergence of Markov chains was monitored using the potential scale reduction factor (i.e., the Gelman-Rubin diagnostic, Rhat), and the effective sample size.

| **Species** | **Count** | **Minimum** | **Median** | **Estimated mean (95% posterior interval)** | **Maximum** | **Effective sample size** | **Rhat** |
| --- | --- | --- | --- | --- | --- | --- | --- |
| **E1** | 2 | 14 | 14 | 13.9 (4.9, 40) | 14 | 4203 | 1.0001 |
| **E2** | 5 | 14 | 14 | 16.9 (8.6, 33.6) | 37 | 4727 | 0.9997 |
| **E3** | 7 | 37 | 84 | 86.6 (50.9, 155.5) | 610 | 4825 | 0.9995 |
| **E4** | 3 | 76 | 114 | 116.7 (47.3, 272.7) | 184 | 5223 | 0.9992 |
| **E5** | 7 | 14 | 30 | 31.7 (18.3, 55.5) | 114 | 4399 | 0.9996 |
| **Ew** | 40 | 37 | 114 | 117.7 (87.6, 141.5) | 610 | 4301 | 0.9997 |
| **Eh6** | 5 | 43 | 76 | 80.9 (41.4, 156.7) | 168 | 4245 | 0.9993 |
| **Eh7** | 1 | 113 | 113 | 112.3 (24.9, 499.3) | 113 | 4301 | 0.9997 |

**Table S7.** Segmented regression analysis of *Bartonella* prevalence, load, and diversity. Coefficients and confidence intervals were estimated for the change in slope at breakpoints. Statistical significance of parameters is indicated with an asterisk based on whether the confidence intervals overlap zero.

| **Regression variable** | **Model family (link)** | **AICc** | **Coefficient** | **Estimate (95% CI)** |
| --- | --- | --- | --- | --- |
| Infection prevalence | Binomial (logit link) | 707.9 | Change1-2 | -0.01 (-0.014, -0.007)* |
|  |  |  | Change2-3 | 0.025 (0.019, 0.031)* |
|  |  |  | Point1-2 | 211.5 (178.5, 244.6) |
|  |  |  | Point2-3 | 833.2 (806, 860.5) |
| Ct value | Gamma (inverse) | 1029.4 | Change1-2 | -0.000038 (-0.000052, -0.000023)* |
|  |  |  | Change2-3 | 0.000053 (0.0000082, 0.000097)* |
|  |  |  | Point1-2 | 216 (171.9, 260.1) |
|  |  |  | Point2-3 | 874.6 (792.7, 956.4) |
| Coinfection prevalence | Binomial (logit link) | 86.2 | Change1-2 | -0.01 (-0.031, 0.01) |
|  |  |  | Change2-3 | 0.015 (0.0071, 0.023)* |
|  |  |  | Point1-2 | 142.6 (-68.9, 354) |
|  |  |  | Point2-3 | 672 (522.8, 821.3) |
| Species richness | Poisson (log) | 65.5 | Change1-2 | -0.0038 (-0.019, 0.011) |
|  |  |  | Point1-2 | 184 (-371.8, 739.8) |
| Shannon number | Gamma (identity) | 44.9 | Change1-2 | 0.0048 (-0.0015, 0.011) |
|  |  |  | Point1-2 | 584 (188, 980) |
| Inv. Simpson index | Gamma (identity) | 39.6 | Change1-2 | 0.0067 (0.0015, 0.012)* |
|  |  |  | Point1-2 | 465 (217.1, 712.9) |
| Species in sample | Poisson (log) | 1107.9 | Change1-2 | 0.0014 (-0.00019, 0.0031) |
|  |  |  | Point1-2 | 584 (217.6, 950.4) |
| Beta diversity | Binomial (logit link) | 11817.4 | Change1-2 | 0.007 (0.004, 0.01)* |
|  |  |  | Change2-3 | -0.014 (-0.023, -0.0052)* |
|  |  |  | Point1-2 | 297 (253.5, 340.5) |
|  |  |  | Point2-3 | 931 (912.4, 949.6) |

**Table S8.** Multinomial and binomial likelihood ratio (LR) tests for changes in *Bartonella* relative species counts before and after the restocking of bat flies. The period before flies were restocked covers July 2009 to July 2011. The period after flies were restocked covers 17 January 2012 to March 2012.

| **Date** | **E1** | **E2** | **E3** | **E4** | **E5** | **Ew** | **Eh6** | **Eh7** |
| --- | --- | --- | --- | --- | --- | --- | --- | --- |
| 2009-07-28 | 0 | 0 | 6 | 3 | 1 | 3 | 0 | 0 |
| 2009-11-05 | 0 | 0 | 5 | 4 | 3 | 2 | 2 | 0 |
| 2010-01-28 | 3 | 3 | 24 | 10 | 12 | 31 | 6 | 3 |
| 2010-03-06 | 3 | 5 | 12 | 15 | 7 | 32 | 3 | 2 |
| 2010-05-21 | 0 | 0 | 3 | 10 | 1 | 24 | 6 | 1 |
| 2010-07-14 | 1 | 1 | 4 | 8 | 4 | 28 | 5 | 1 |
| 2010-09-23 | 0 | 0 | 4 | 2 | 12 | 23 | 8 | 0 |
| 2010-11-05 | 1 | 2 | 4 | 2 | 5 | 27 | 6 | 0 |
| 2011-03-04 | 0 | 0 | 2 | 1 | 0 | 4 | 3 | 0 |
| 2011-07-13 | 3 | 0 | 2 | 3 | 2 | 9 | 1 | 0 |
| 2012-01-17 | 6 | 6 | 7 | 2 | 11 | 6 | 0 | 0 |
| 2012-01-31 | 8 | 8 | 8 | 0 | 12 | 11 | 0 | 0 |
| 2012-02-14 | 7 | 3 | 5 | 4 | 13 | 9 | 0 | 0 |
| 2012-03-15 | 2 | 5 | 17 | 0 | 16 | 11 | 1 | 0 |
| Total counts | 34 | 33 | 103 | 64 | 99 | 220 | 41 | 7 |
| Sum total counts | 601 |  |  |  |  |  |  |  |
| Before counts | 11 | 11 | 66 | 58 | 47 | 183 | 40 | 7 |
| Before total counts | 423 |  |  |  |  |  |  |  |
| Before frequency | 0.026 | 0.026 | 0.16 | 0.14 | 0.11 | 0.43 | 0.095 | 0.017 |
| After counts | 23 | 22 | 37 | 6 | 52 | 37 | 1 | 0 |
| After total counts | 178 |  |  |  |  |  |  |  |
| After frequency | 0.13 | 0.12 | 0.21 | 0.034 | 0.29 | 0.21 | 0.0056 | 0 |
| Multinomial adjusted LR | 183.3 |  |  |  |  |  |  |  |
| Multinomial P | 0 |  |  |  |  |  |  |  |
| Binomial adjusted LR | 38.2 | 34.9 | 3.3 | 21.6 | 42.2 | 39 | 26.9 | 5.8 |
| Binomial P | 6.4E-10 | 3.5E-09 | 0.07 | 3.3E-06 | 8.2E-11 | 4.2E-10 | 2.1E-07 | 0.016 |

**Table S9.** Multinomial and binomial likelihood ratio (LR) tests of *Bartonella* species count changes between groups of sampled bats and bat flies: (A) tests between captive bats and captive flies in March 2010, (B) tests between wild bats and wild flies on 17 January 2012, and (C) tests between captive bats (aggregated over the period after flies were restocked) and sampled wild flies on 17 January 2012.

| **A** |  |  |  |  |  |  |  |  |  |
| --- | --- | --- | --- | --- | --- | --- | --- | --- | --- |
| **Date** | **Group** | **E1** | **E2** | **E3** | **E4** | **E5** | **Ew** | **Eh6** | **Eh7** |
| 2010-03-06 | captive bats | 3 | 5 | 12 | 15 | 7 | 32 | 3 | 2 |
| 2010-03-06 | captive flies | 0 | 1 | 2 | 0 | 7 | 15 | 0 | 1 |
| Bats total counts |  | 79 |  |  |  |  |  |  |  |
| Bats frequency |  | 0.023 | 0.045 | 0.14 | 0.13 | 0.083 | 0.55 | 0.023 | 0.015 |
| Flies total counts |  | 26 |  |  |  |  |  |  |  |
| Flies frequency |  | 0 | 0.038 | 0.077 | 0 | 0.27 | 0.58 | 0 | 0.038 |
| Multinomial adjusted LR |  | 14.9 |  |  |  |  |  |  |  |
| Multinomial P |  | 0.038 |  |  |  |  |  |  |  |
| Binomial adjusted LR |  | 1.0 | 0.023 | 0.85 | 6.1 | 6.6 | 0.06 | 1.0 | 0.57 |
| Binomial P |  | 0.31 | 0.88 | 0.36 | 0.013 | 0.01 | 0.8 | 0.31 | 0.45 |
|  |  |  |  |  |  |  |  |  |  |
| **B** |  |  |  |  |  |  |  |  |  |
| **Date** | **Group** | **E1** | **E2** | **E3** | **E4** | **E5** | **Ew** | **Eh6** | **Eh7** |
| 2012-01-17 | wild bats | 1 | 5 | 21 | 6 | 17 | 23 | 5 | 6 |
| 2012-01-17 | wild flies | 0 | 1 | 5 | 2 | 6 | 6 | 0 | 0 |
| Bats total counts |  | 84 |  |  |  |  |  |  |  |
| Bats frequency |  | 0.012 | 0.06 | 0.25 | 0.071 | 0.20 | 0.27 | 0.06 | 0.071 |
| Flies total counts |  | 20 |  |  |  |  |  |  |  |
| Flies frequency |  | 0 | 0.05 | 0.25 | 0.1 | 0.3 | 0.3 | 0 | 0 |
| Multinomial adjusted LR |  | 5.8 |  |  |  |  |  |  |  |
| Multinomial P |  | 0.57 |  |  |  |  |  |  |  |
| Binomial adjusted LR |  | 0.41 | 0.03 | 0 | 0.19 | 0.91 | 0.06 | 2.1 | 2.5 |
| Binomial P |  | 0.52 | 0.86 | 1 | 0.67 | 0.34 | 0.81 | 0.15 | 0.11 |
|  |  |  |  |  |  |  |  |  |  |
| **C** |  |  |  |  |  |  |  |  |  |
| **Date** | **Group** | **E1** | **E2** | **E3** | **E4** | **E5** | **Ew** | **Eh6** | **Eh7** |
| 2012-01-17 | captive bats | 34 | 34 | 53 | 8 | 99 | 57 | 1 | 0 |
| 2012-01-17 | wild bats | 1 | 5 | 21 | 6 | 17 | 23 | 5 | 6 |
| Captive bats total counts |  | 286 |  |  |  |  |  |  |  |
| Captive bats frequency |  | 0.12 | 0.12 | 0.19 | 0.028 | 0.35 | 0.2 | 0.0035 |  |
| Wild bats total counts |  | 84 |  |  |  |  |  |  |  |
| Wild bats frequency |  | 0.013 | 0.064 | 0.27 | 0.077 | 0.22 | 0.29 | 0.064 |  |
| Multinomial adjusted LR |  | 43.3 |  |  |  |  |  |  |  |
| Multinomial P |  | 1.0E-07 |  |  |  |  |  |  |  |
| Binomial adjusted LR |  | 11.6 | 2.3 | 2.9 | 4.2 | 5.4 | 3.6 | 17.7 |  |
| Binomial P |  | 6.5E-04 | 0.12 | 0.087 | 0.041 | 0.02 | 0.058 | 2.5E-05 |  |
