# Supplementary figures and images for: Manipulating vector transmission reveals local processes in Bartonella communities of bats
Source: Parasitology. 2026 Feb 2;153(3):420–31. doi: 10.1017/S0031182026101656 (PMC13215733; doi:10.1017/S0031182026101656)

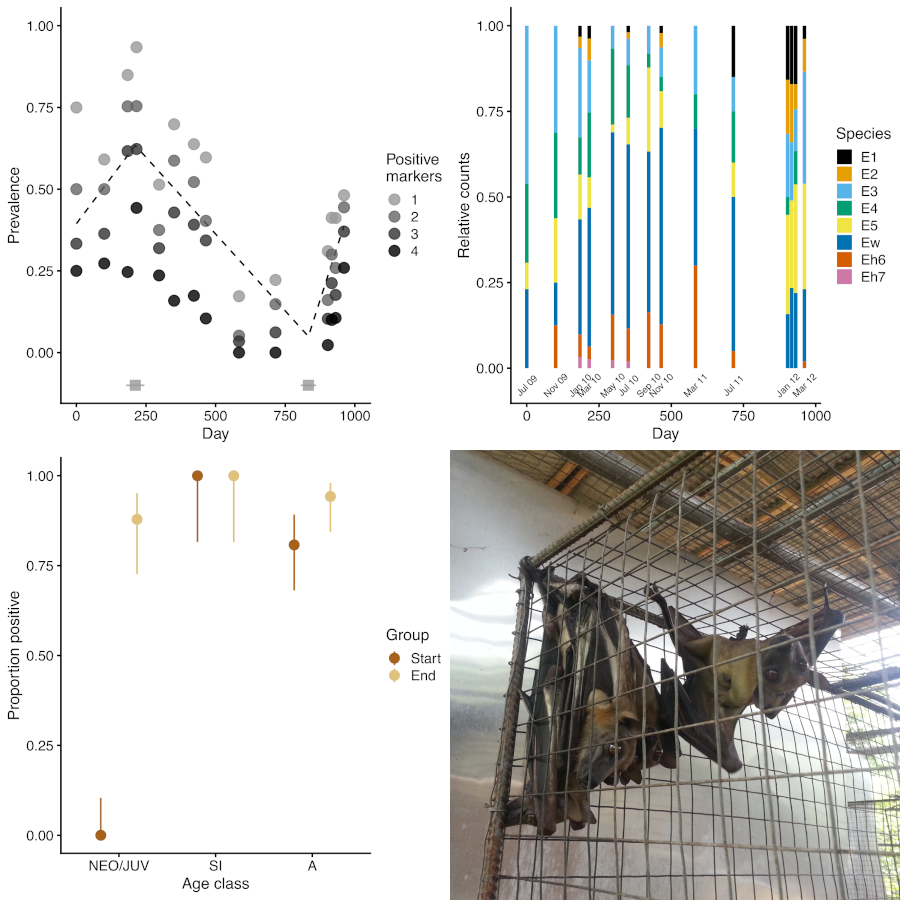

Supplement: McKee et al. supplementary material 2 — McKee et al. supplementary material [file S0031182026101656sup002.png]
